# Exploring the diversity of AVPR2 in Primates and its evolutionary implications

**DOI:** 10.1590/1678-4685-GMB-2023-0045

**Published:** 2023-11-03

**Authors:** Bibiana Sampaio de Oliveira Fam, Pedro Vargas-Pinilla, Pâmela Paré, Luane Landau, Lucas H. Viscardi, Alcides Pissinatti, Tiago Falótico, Renan Maestri, Maria Cátira Bortolini

**Affiliations:** 1 Universidade Federal do Rio Grande do Sul, Departamento de Genética, Laboratório de Evolução Humana e Molecular, Porto Alegre, RS, Brazil.; 2 Universidade de São Paulo, Faculdade de Medicina, Departamento de Bioquímica e Imunologia, Ribeirão Preto, SP, Brazil.; 3 Universidade de São Paulo, Escola de Artes, Ciências e Humanidades, São Paulo, SP, Brazil.; 4 Universidade Federal do Rio Grande do Sul, Departamento de Ecologia, Laboratório de Ecomorfologia e Macroevolução, Porto Alegre, RS, Brazil.; 5 Centro de Primatologia do Rio de Janeiro, Rio de Janeiro, RJ, Brazil.

**Keywords:** AVPR2, diversity, co-evolution, Primates

## Abstract

The current study focuses on the investigation of AVPR2 (VTR2C) protein-coupled receptor variants specific to different primate taxa. AVPR2 is activated by the neurohormone AVP, which modulates physiological processes, including water homeostasis. Our findings reveal positive selection at three AVPR2 sites at positions 190, 250, and 346. Variation at position 250 is associated with human Congenital Nephrogenic Diabetes Insipidus (cNDI), a condition characterized by excessive water loss. Other 13 functional sites with potential adaptive relevance include positions 185, 202, 204, and 252 associated with cNDI. We identified SH3-binding motifs in AVPR2’s ICL3 and N-terminus domains, with some losses observed in clades of Cercopithecidae, Callitrichinae, and Atelidae. SH3-binding motifs are crucial in regulating cellular physiology, indicating that the differences may be adaptive. Co-evolution was found between AVPR2 residues and those in the AVP signal peptide/Neurophysin-2 and AQP2, other molecules in the same signaling cascade. No significant correlation was found between these Primates’ taxon-specific variants and the bioclimatic variables of the areas where they live. Distinct co-evolving amino acid sequences in functional sites were found in Platyrrhini and Catarrhini, which may have adaptive implications involving glucocorticoid hormones, suggesting varied selective pressures. Further studies are required to confirm these results.

## Introduction

### AVPR2 receptor and its function 

The neurohormone arginine vasopressin (AVP or VT in the newly proposed nomenclature (Theofanopoulou et al., 2021) and its paralog, the neurohormone oxytocin (OXT or OT; Theofanopoulou *et al.*, 2021), are the main ligands of the oxytocinergic system, which plays a decisive role in the behavior and physiology of vertebrates. For example, AVP acts on physiological salt and water balance (Seibold et al., 1992; Ocampo Daza et al., 2012; Wacker and Ludwig, 2019), and this function is primarily dependent on its binding to the vasopressin receptor AVPR2 (or VTR2C; Theofanopoulou *et al.*, 2021), a Class-A G protein-coupled receptor (GPCR). Extracellular signals from GPCRs promote structural rearrangements in their cytoplasmic regions, which are then recognized by transducer G proteins and regulate intracellular messengers (Yang et al., 2018).

The human canonical AVPR2 isoform (NM_000054) has 371 amino acids (Birnbaumer et al.,1992; Lolait et al., 1992). Similar to other GPCRs, AVPR2 has seven transmembrane domains (TM1-7), an N-terminus domain and three loops in the extracellular portion (N-terminus; ECL1-3), a C-terminal domain, and three loops in the cytoplasmic/intracellular region (ICL1-3; C-terminal) (Birnbaumer *et al.*,1992). 

AVPR2 is mainly expressed at the basolateral membrane of kidney cells, but its presence in other organs and tissues, such as the pituitary gland, has been reported (Dahia et al., 1996; Wang et al., 2012; Bous et al., 2021). The complex AVP-AVPR2 activates one G protein subtype (Gs) of the adenylyl cyclase, which increases intracellular cyclic adenosine monophosphate (cAMP) (Schöneberg et al., 1998; Bous *et al.*, 2021). Increased levels of cAMP enhance the activity of basophilic protein kinases, including protein kinase A (PKA) (Bous *et al.*, 2021). While AVPR2 modulates the signaling via Gs and cAMP, the other known receptors of the oxytocinergic system are mainly transcribed in the brain (OXTR, AVPR1a, and AVPR1b; or OTR, VTR1A, and VTR1B; Theofanopoulou et al., 2021) maintain the molecular signaling cascade through Gq/11 (Meyerowitz et al., 2022) and with Ca^2+^ release (Lolait et al., 1992; Lee et al., 2009). AVPR2 is also independent of the magnesium ion (Mg^2+^) level for its activation, while OXTR, AVPR1a, and AVPR1b are Mg^2+^ dependent (Meyerowitz *et al.*, 2022). Another difference is a shorter N-terminus domain, which confers differentiated AVPR2-Gs protein coupling selectivity (Holmes et al., 2003). 

The AVP-AVPR2 complex stimulates the increase of cAMP levels, which in turn activates PKA and leads to the phosphorylation of Aquaporin 2 (AQP2) at specific sites. This results in the accumulation of AQP2 on the apical cell membrane of the collecting duct in the kidney (Bous et al., 2021; Olesen and Fenton, 2021). While this pathway does not entirely explain AQP2 plasma membrane targeting, it is widely recognized as critical for AQP2’s tetramer formation into channels that make the cell membrane permeable. This structure allows for the passage of ions and water molecules, ultimately regulating urine concentration and blood pressure (Deen et al., 1994; Schöneberg et al., 1998; Koshimizu et al., 2012; Wacker and Ludwig, 2019; Bous *et al.*, 2021). In other words, the activation of AVPR2 by AVP plays a crucial role in regulating AQP2 in both the short and long term. This complex mechanism provides a sophisticated physiological response, ensuring that vertebrates can properly balance salt levels in their bodies. For example, animals living in freshwater environments must actively take up salts, while those in seawater must excrete excess salts (Schrier, 2008; Wacker and Ludwig, 2019; Olesen and Fenton, 2021). Besides, under conditions where a stress response is expected, not only AVPR1b is transcribed, but AVPR2, both modulating the mouse hypothalamic-pituitary-adrenal (HPA) axis (with sex-dependent mechanisms), which stimulates the release of cortisol and other glucocorticoids (Taheri et al., 2022).

Böselt et al. (2009) discovered through their study of various marsupial species that they possess a deletion of 11 amino acids at positions 243-253 of the ICL3 of AVPR2, compared to the human AVPR2 ICL3. Their functional experiments showed that these structural changes in the marsupial AVPR2 improved function, which could provide an advantage for maintaining water and electrolyte balance in arid environments. However, there is no evidence of a single amino acid being responsible for causing constitutive activity upon substitution. The authors suggest that it is more likely that sequential or combined changes in the marsupial AVPR2 contribute to enhanced basal receptor function (Böselt *et al.*, 2009). 

### Adaptation in Primates

Like other animals, primates must maintain a tight balance of water gain and loss each day, but with noticeable differences (Cheuvront et al., 2010). For example, Pontzer et al. (2021) compared isotope-depletion measures of water turnover in zoo- and rainforest-sanctuary-housed apes (chimpanzees, bonobos, gorillas, and orangutans) with humans from five distinct populations, including hunter-gatherers living in a semi-arid savannah. Humans drink daily to maintain water balance, unlike rainforest-living great apes, which obtain water from their food. Apes can go days or weeks without drinking (Pontzer *et al.*, 2021). They found that water turnover was strongly related to total energy expenditure, physical activity, fat-free mass, and ambient temperature and humidity, at least considering Old World primates (OWp; parvorder Catarrhini) that they studied. In analyses controlling for those factors, water turnover was 30-50% lower in humans than in great apes despite humans’ greater sweating capacity (Pontzer *et al.*, 2021). According to the authors, lower water turnover and water/energy ratio in humans suggest natural selection to conserve water in the hominin lineage. Besides, dietary changes, particularly cooking, increased caloric density and reduced the water content of hominin foods relative to other primates, including apes (Pontzer *et al.*, 2021). 

The genetic mechanisms underlying these adaptations in Primates species are not completely comprehended at present. However, studies focusing on human diseases have provided insights into the involvement of AVP, AVPR2, and AQP2. It has been discovered that disruptions in the AVP-AVPR2-AQP2 axis can result in water balance disorders in humans, including conditions like hyponatremia caused by congestive heart failure, hypertension, or hepatic cirrhosis, as well as urinary problems like incontinence and nocturia (Ball, 2007; Bous et al., 2021).

Diabetes Insipidus, characterized by excessive water loss, is one of the best studied diseases in this context. Approximately 250 mutations in the AVPR2 gene have been found to explain nearly 90% of human Congenital Nephrogenic Diabetes Insipidus (cNDI), with the remaining 10% attributed to mutations in AQP2 (Bichet and Bockenhauer, 2016; Peng et al., 2019; Li et al., 2021). As a result, collecting ducts do not reabsorb water as they should. Thus, cNDI leads to a failure to reabsorb water in collecting ducts and results in chronic excessive thirst, excessive urine production, and potentially severe dehydration and mental retardation despite appropriate AVP secretion (Ando and Uchida, 2018). On the other hand, Central Diabetes Insipidus (CDI) is characterized by hypotonic polyuria due to an impairment of AVP secretion. Familial CDI is primarily inherited in an autosomal dominant manner, and around 80 causal mutations in the AVP gene have been reported (Arima et al., 2016).

Noteworthy, the complete AVP gene (or AVP-NPII) codes for the neurohormone AVP as well as the signal peptide, which plays a critical role in protein targeting and translocation, the Neurophysin-2 (a precursor and carrier protein for AVP), and a glycoprotein, Copeptin, whose function remains unknown. Mutations in the AVP affecting signal peptide, the hormone, and the Neurophysin-2 were associated with familial CDI (García-Castaño et al., 2020). So far, no causative mutation of CDI was found located in Copeptin (García-Castaño *et al.*, 2020). 

These types of results, derived from human disease studies, provide a valuable starting point for exploring the connection between variations within and between primate species and specific evolutionary adaptations.

Our objective is to provide an overview of the genetic diversity of the AVPR2 coding region by using both existing data from the literature and original data from 38 species of New World primates (NWp; parvorder Platyrrhini). Our study investigated the correlation between this genetic diversity and the environmental conditions, such as temperature and precipitation, in the habitats of these primates. 

We also examined the taxon-specific AVPR2 amino acids compared to those of other correlated molecules, such as AQP2 and AVP/signal peptide/Neurophysin-2, to see if they could be co-evolving. These are some of the analyses we have performed.

## Material and Methods

### Primate sampling and sequencing of AVPR2 

The current project is registered in the Biodiversity Authorization and Information System (SISBIO; protocol numbers; 48323-1, 05/05/2015, 57039, 09/01/2017; and 59019-1, 23/06/2017) and SISGEN (National System for the Management of Genetic Heritage and Associated Traditional Knowledge; protocol number AF00ED5; 27/09/2018). The State Environmental Institute (INEA), the Animal Ethics Committees of the “Universidade Federal Rio Grande do Sul” and the “Universidade de São Paulo” approved the project. Our studies with these samples comply with the principles proposed by the American Society of Primatologists for the ethical treatment of non-human primates (https://www.asp.org/society/resolutions/ethicaltreatmentofnonhumanprimates.cfm).

Blood or fecal material from individuals of 38 Platyrrhini species (SI Appendix, Table S1) were collected in the “Rio de Janeiro” Primatology Center (CPRJ) and “Serra da Capivara” National Park (SCNP), respectively. DNA extraction was performed using Qiagen DNeasy Blood and Tissue Kit® protocol according to the manufacturer’s instructions. Primer sets were designed to flank whole coding regions of AVPR2 (SI Appendix, Table S2). The amplification process (PCR) begins with an initial denaturation step at 94 °C for 5 minutes. This is followed by 35 cycles of amplification, each consisting of 30 seconds at 94 °C, 30 s at the annealing temperature specific to each primer, and an extension step of 45 s at 72 °C. After the cycles, a final extension is performed for 10 min at 72 °C. The success of the DNA extraction was confirmed by visually observing the DNA under a UV light on a 1% agarose gel that was stained with a DNA dye called GelRedTM. The concentration of the DNA samples was measured using a spectrophotometer called NanoDrop® from Uniscience. During electrophoresis, a low-mass molecular marker (100 bp) was used as a reference to track the movement of the DNA fragments. The sequences were obtained through an external service provider (Macrogen). Sequences were aligned using the MUSCLE (Edgar, 2004) algorithm implemented in Aliview software (Larsson, 2014). 

### Evolutionary analysis of the Primates AVPR2

The AVPR2 coding sequences data set includes 45 Platyrrhini, 25 Catarrhini, and 6 Strepsirrhini species, plus two outgroup species (order Scandentia and Dermoptera), which are considered most likely sister clades in relation to Primates (SI Appendix). Two different approaches are used to estimate evolutionary rates using the codeml program in PAML v4.9 (Yang, 2007) and Mixed Effects Model of Evolution (MEME; Murrell et al., 2012) implemented in Hyphy (www.hyphy.org; Pond et al., 2005). We established a p-value ≤ 0.1 for MEME for statistical significance thresholds (Spielman et al., 2019).

### Short linear motif prediction of Primates AVPR2

We predicted the existence of Short Linear Motifs (SLiMs) using the Eukaryotic Linear Motif (ELM) web server (http://elm.eu.org). ELM is the most comprehensive repository of experimentally validated SLiMs (Dinkel et al., 2014; Van Roey et al., 2014). Since these predictions can introduce false positives (Teyra et al., 2017), we only considered SLiMs in disordered regions (IDRs) of the proteins, supported by experimental evidence in ELM. The primary feature of IDRs is the ability to assume different conformations (*i.e*., with no fixed tertiary structure) that allow interaction with multiple partners (Uversky, 2015). We just considered SLiMs with the probability of them being found at random ≤ 6%.

### Climatic and ecological data vs. Primates taxon-specific AVPR2 variants

The 76 Primates species with available genetic data were analyzed according to their geographic distribution. Environmental variables of the regions where the species are geographically distributed were also obtained from the IUCN database (https://www.iucnredlist.org/). Bioclimatic variables were obtained through the Worldclim database (http://www.worldclim.com/version2) with a 30’ resolution (1 km^2^). From the spatial distribution, the average of each one of the 19 bioclimatic variables was estimated for each of the analyzed species (Table S3) using rgeos (v.0.5-9; Bivand, 2021) and rgdal (v.1.5-28, Bivand, 2021) packages in the R environment. 

The multivariate phylogenetic comparative methods to analyze the level of correlation between the climatic/ecological data and relevant AVPR2 sites were performed. The Phylogenetic Partial Least Square (pPLS) analysis, a test based on a covariance matrix, with accounting phylogenetic relationship among taxa (Adams and Felice, 2014), was implemented in the R environment with geomorph package (v.4.0.1; Baken et al., 2021). 

### Co-evolution analyses 

We evaluated the co-evolution process of these molecules in Primates species available in public databases through the Fastcov (Shen and Li, 2016) (Tables S1, S3, and S4). We considered covariant sites with a degree ≥ 0.95.

The correlation of the co-evolving amino acids con-sidering AVP/signal peptide/Neurophysin-2, AVPR2, and AQP2 with the climatic/ecological data was tested using the same procedures described in the previous item.

The file Data 1 in Supplementary Material provides a complete description of the methods employed and the corresponding references.

ChatGPT was used to improve English writing. 

## Results

### Evolutionary analysis of the Primates AVPR2

**Figure 1 -:**
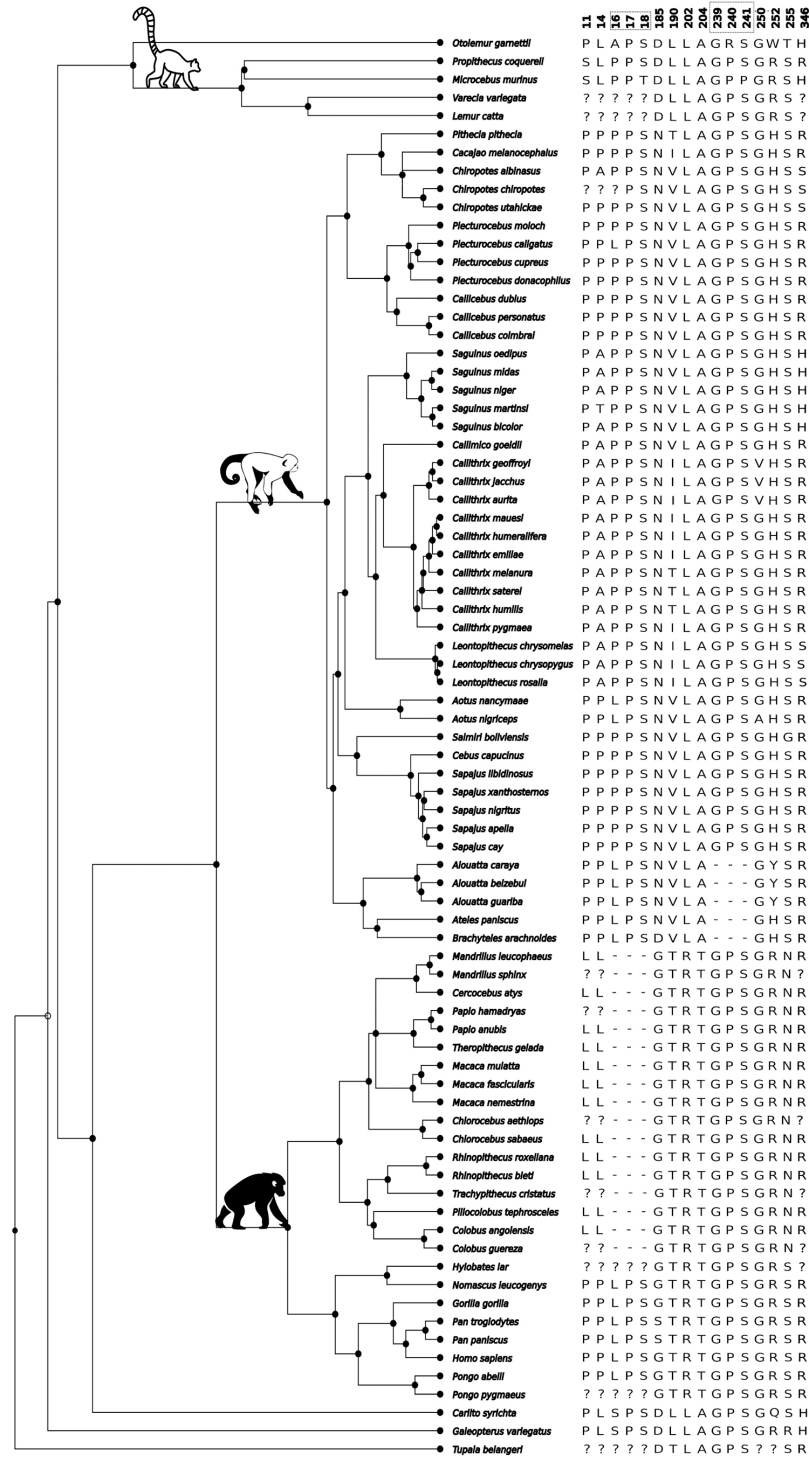
Schematic representation of taxon-specific Primates AVPR2 variants with potential functional and evolutionary relevance according to the present study: Sites with a high probability of being under positive selection (190, 250, 346); Indels identified at positions 16-18 and 239-241 (in the square); change in the composition of prolines (P) at positions 11 and 14 in the AVPR2 N-Terminal domain; phosphorylation site at position 255; mutations at position 185, 202, 204, 250 and 252 are part of the cNDI repertoire; Bichet et al., 1994; Bichet, 1998; Postina et al., 2000; Spanakis et al., 2008; https://www.uniprot.org/uniprotkb/P30518/entry).

The best-fitting model for the data was the M8 model of the PALM analysis, which indicated positive selection at some sites (ω= 2.62, p = 0.0008; Table S6, Figure 1, Figure S1). AVPR2 in Primates was found to have 98% of its sites under negative selection, 1% under neutrality, and 1% under positive selection. This result suggests that the neutral model cannot explain the amino acid variability in some sites across species, and positive selection may have played an essential role. The highest probability of positive selection was observed at site 190 (BEB = 0.99). The MEME test, another codon-level method better than PALM at detecting episodic positive selection, also showed a positive selection signature (Table S7). In addition to site 190 (*p* = 0.03), MEME detected positive selection at sites 250 (*p* = 0.06) and 346 (*p* = 0.08). Of note, the change from glycine (G) to valine (V) at site 250 is only present in a specific branch of Callithrix, indicating a potential episodic positive selection case. 

The positions 190, 250, and 346 (Figure 1) in *Homo sapiens*, the most investigated Primates species do not show significant polymorphisms according to searches in public databases. This finding suggests that the observed amino acid residues are taxon-specific and characterize taxonomic groups, such as species, genus, family, or order. Consequently, diversity within species (at the population level) may not be significant. However, it could still have relevance on an individual or family basis, as in the case of mutations at position 250 of AVPR2, associated with cNDI.

Figure 1 shows 13 taxon-specific variants of Primates AVPR2 with potential functional and evolutionary significance. For example, mutations at positions 185, 202, 204, 250, and 252 are part of the cNDI repertoire (Bichet et al., 1994; Bichet, 1998; Postina et al., 2000; Spanakis et al., 2008; https://www.uniprot.org/uniprotkb/P30518/entry). We also identified insertions/deletions (indels) at positions 16-18 and 239-241 in the N-terminus (residues 1 to 38) and ICL3 (221-271) domains, respectively. The loss of prolines (P) at positions 11 and 14 and changes in the serine (S) phosphorylation site at position 255 (Wu et al., 2008) also in the N-terminus and ICL3 domains, respectively, in some clades are noticeable. For example, in the Cercopithecidae species, the S is replaced by an asparagine (N). Previous sequence analysis showed that the 255-S allele is conserved in the VPR2 orthologs of mice, rats, cattle, dogs, pigs, and horses (Wu *et al.*, 2008).

### Short linear motif prediction of the Primates AVPR2

We predicted the presence of SLiMs in AVPR2 (Table S8, Figure S2) as these molecular elements are crucial for the dynamic and proper control of cellular physiology (Van Roey et al., 2014). 

Under certain conditions (described in the Materials and Methods section), three overlapping short linear motifs (SLiMs) were predicted in AVPR2 at positions 8-14, 11-17, and 14-20, with the canonical pattern +xX[P]xXP (where P is a proline, X is usually a hydrophobic residue, and x is any residue). These SLiMs are located in the N-terminus domain of AVPR2, which recognizes the SRC Homology 3 (SH3), a small protein domain of approximately 60 amino acids. The XP dipeptides occupy two hydrophobic pockets in the SH3 ligand-binding groove, while a third slot contacts additional residues on either side of the motif (Aitio et al., 2010). 

Another SLiM at positions 234-240 was identified in the ICL3 domain, characterized by a different pattern from the canonical motif due to a valine residue (xxx[V]xxP). According to the ELM database, this SLiM was identified as a non-canonical SH3-binding motif. 

Our findings show a loss of SLiMs in the parvorders Catarrhini and Platyrrhini due to deletions. A deletion at positions 16-18 in the N-terminus was found in all species of Cercopithecidae, while another deletion at positions 239-241 in ICL3 was found in Atelidae species, resulting in the loss of predicted SH3- binding sites.

We also found that the proline (P) residues at positions 11 and 14 were replaced by leucine (L) in Cercopithecidae species, and the P at position 14 was replaced by alanine (A) in Callitrichinae species. The replacement of P led to the loss of the predicted N-terminus SLiMs that recognize the SH3 in all Cercopithecidae and Callitrichinae species.

These results reveal a remarkable diversity in the predicted SH3-binding sites across the Primates taxon in both the N-terminus and ICL3 domains of AVPR2. 

### Co-evolution analysis

Since the AVP-AVPR2-AQP2 system plays a critical role in maintaining fluid homeostasis in mammals, we searched for evidence of co-evolution between these molecules (*i.e*., not random inter-residue correlations in individual proteins). In this study, we chose to examine all three molecules together rather than in pairs.

The molecular co-evolution analyses showed interesting points of inter-molecular co-variation considering a threshold of association degree of correlation ≥0.95 for sites in co-evolution (Table S9). We found ten sites in AVPR2 co-evolving with the two other molecules (AVP, complete coding sequence, and AQP2). As the nine amino acids (C-Y-F-G-N-C-P-R-G) of the neurohormone, AVP is conserved in most placental mammals, including Primates, the sites in co-evolution with AQP2 and/or AVPR2 are located at the signal peptide and in the Neurophysin-2, both molecules encoded by the *AVP* gene. 

**Figure 2 -:**
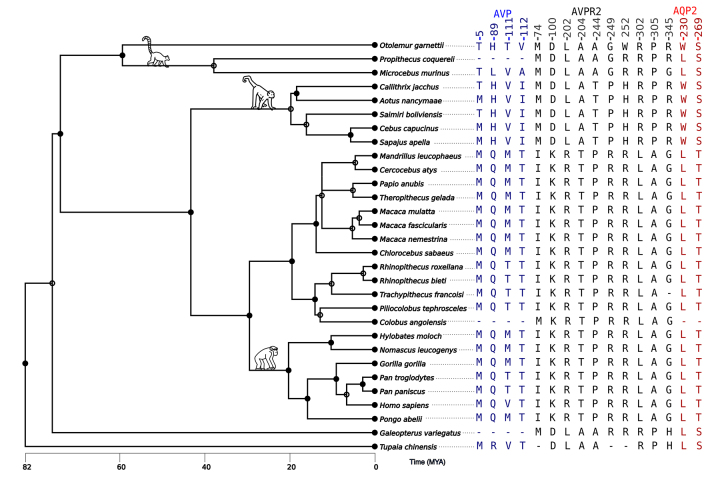
The AVP, AVPR2, and AQP2 co-evolution sites with an occurrence probability ≥0.95.

Figure 2 illustrates the amino acids with potential evolutionary interdependence. Signs of co-evolution were found in 4 positions of AVP, one at position 5 (signal peptide) and three at positions 89, 111, and 112 of the Neurophysin-2 molecule. Two sites in AQP2, at positions 230 and 269 in the C-terminal cytoplasmic region, are co-evolving with the other molecules. In AVPR2, a total of ten sites are found: one in ICL1 (position 74), one in ECL1 (100), three in TM5 (202, 204, and 220), three in ICL3 (244, 249, and 252), two in ECL3 (302 and 305), and one in C-terminal (345).

Table S9 and Figure 2 illustrate the blocks of specific combinations of amino acids in AVPR2 that are statistically expected, based on the combination of amino acids found in the AVP signal peptide/Neurophysin-2 and AQP2. These blocks vary across different taxonomic groups. For example, the amino acid pattern (AVP signal peptide/Neurophysin-2: 5-T/M 89-H 111-V 112-I; AVPR2: 74-M 100-D 202-L 204-A 244-T 249-P 252-H 302-R 305-P 345-R; AQP2: 230-W and 269-S) distinguished Platyrrhini clade sequences from Catarrhini clade sequences (signal peptide/Neurophysin-2: 5-M 89-Q 111-V/T/M 112-T; AVPR2: 74-I/M 100-K 202-R 204-T 244-P 249-R 252-R 302-L 305-A 345-G; AQP2: 230-L and 269-T) (Table S9; Figure 2). 

### Climatic and ecological data vs Primates taxon-specific AVPR2 variants

The multivariate phylogenetic comparative methods to analyze the level of correlation were performed using two datasets: the first set considered the Primates AVPR2 sites with potential functional and evolutionary relevancies (Figure 1). The amino acids at positions 16-18 and 239-241 were not considered independent in the analysis but as blocks, *i.e.*, the presence or absence of them (or indels). 

The phylogenetic Partial Least Squares analysis did not attain a significant correlation considering the block with the AVPR2 genetic variables (Figure 1) and the 19 climatic variables (r-PLS: 0.202, p = 0.534; Figure S3A). 

Since some of these 19 climate variables are not independent, we also generated a PCA analysis and compared the two main components with the genetic data from Figure 1. Variables related to temperature are prevalent in the first principal component (PC1, which explains 52% of the total variance). In contrast, the second principal component (PC2, explains 19% of the total variance and has precipitation seasonality as the primary climatic variable) (Table S5). No significant correlation was found (r-PLS: 0.186, p = 0.667; Figure S3B).

Other analyses involved the block of ten AVPR2 sites in co-evolution with AVP and AQP2 (Figure S2), the 19 bioclimatic variables, or the PC1 and PC2. No significant correlation was found (r-PLS: 0.344, p = 0.09; and r-PLS: 0.097, p = 0.99; Figures S4A and S4B, respectively).

## Discussion

A successful initial strategy to identify potential taxon-specific amino acids is to compare orthologous proteins whose function is well-known and whose related phenotypes are diverse among the investigated taxonomic groups. In the present study, we use this strategy and consider the AVPR2 orthologous in Primates species, a known GPCR receptor responsible for the essential biological function of regulating homeostasis and water balance. 

We could not detect relevant polymorphism regarding the sites (Figure 1, Figures S2, S3, and Table S9) in the most studied species of Primates (*Homo sapiens*) in the literature and public databases, which reinforces the idea that they are taxon-specific (*i.e*., amino acid are fixed in the taxonomic group) due to negative selection and poor tolerance of intra-specific mutation. However, some rare amino acid changes in these sites are responsible for human diseases, such as the AVPR2 positive selected site 185, which has notable variation among Primates branches and recognized functional importance as part of the cNDI genetic repertoire (Bichet, 1998; https://www.uniprot.org/uniprot/P30518). The other variants also follow the taxon-specific pattern.

We also predicted canonical and potential non-canonical SH3 binding motifs in the ICL3 and N-terminus domains, but some branches have lost them (Cercopithecidae, Callitrichinae, and Atelidae species). The relevance of SH3-binding sites, particularly the PxxP motif, located in both N-terminus (Aitio et al., 2010; Lau et al., 2016) or cytoplasmic/intracellular domains (Sun and Soutar, 2003; Magalhaes et al., 2012) has been reported in the literature. For example, Lau *et al.* (2016) found N-terminus binding site(s) for SH3 located in a protein (Tau) that regulates synaptic functions in association with Fyn kinase, which plays an essential role during myelination. Changes to the Tau-Fyn/SH3 interactions play a pathogenic role in neurodegenerative disorders (Lau *et al.*, 2016). A comprehensive search showed that half of GPCRs present SH3-binding PxxP motifs (Oldenhof et al., 1998). The removal of the SH3-binding motifs located at the ICL3 of the D4 dopamine receptor, a class-A GPCR, results in a constitutively internalized receptor, which may account for the deficit in cell signaling (Magalhaes *et al.*, 2012). 

The presence of this kind of molecular structure (*e.g*., SH3-binding PxxP motifs) leads to an unstructured conformation in the ICL3 and N-terminus domains, which increases the plasticity of GPCR IDRs. Studies show that AVPR2 has the highest IDR content in both ICL3 and N-terminus domains when compared with other OXT-AVP receptors in placental mammals (Paré et al., 2016). The loss of a fixed tertiary structure allows for interactions with multiple ligands and other receptors, as Tovo-Rodrigues et al. (2014) demonstrated.

Covariant amino acid changes are crucial for maintaining a protein's structural characteristics and, consequently, conformational and functional stability throughout evolution (Gloor et al., 2005). This concept is also applied to different proteins that are part of complex genetic networks or systems, whose effective action of one molecule is dependent on the effective action of another. As a result, these interacting proteins co-evolve at multiple interconnected scales, from residue-residue, to protein-protein, to family-family level (Szurmant and Weigt, 2018). Our study found indications of co-variation between amino acids of AVPR2, the signal peptide/Neurophysin-2 (encoded by AVP), and AQP2. A residue at a specific position in AVPR2 determines the presence of the other residues at specific positions in the other two molecules.

The combinations of co-evolving amino acids are different when Platyrrhini and Catarrhini are compared. We found no study showing significant differences in water balance between the Catarrhini and Platyrrhini clades. However, there are noticeable differences in cortisol and other glucocorticoid hormones. For instance, Platyrrhini have higher plasma cortisol concentrations and increased urinary free-cortisol excretion compared to Catarrhini (Chrousos et al., 1982). The authors compared this natural state in Platyrrhini to Cushing syndrome, a human condition caused by excessive cortisol exposure, which can lead to increased urination, high blood pressure, insulin resistance, and diabetes. Despite having higher cortisol levels, Platyrrhini monkeys do not experience any metabolic or psychological problems, indicating evolutionary adaptation (Chrousos *et al.*, 1982). 

The gene AVPR2 has been found to be overexpressed in Cushing syndrome and corticotropic tumors, affecting hormone levels and leading to changes in cell growth and function (Dahia et al., 1996; Wang et al., 2012; Fukuoka et al., 2020). Additionally, cortisol helps regulate water balance by increasing AQP2 expression (Wang *et al.*, 2012). Under conditions where a stress response is expected, AVPR2 is transcripted, suggesting a role in the modulation of the hypothalamic-pituitary-adrenal (HPA) axis (with sex-dependent mechanisms), which stimulates the release of cortisol (Taheri et al., 2022). Here we suggested that the different combinations of co-evolving amino acids between these two Primates clades may have adaptive significance regarding the modulation of glucocorticoid hormones. 

It is known that changes at positions 202, 204, and 252 of AVPR2, which co-evolve, are critical functional sites in modulating homeostasis and are part of the cNDI repertoire in the presence of specific mutations (Bichet et al., 1994; Bichet, 1998; Postina et al., 2000; Spanakis et al., 2008; https://www.uniprot.org/uniprotkb/P30518/entry). *In vitro* experiments show that a receptor with a cysteine (C) at position 202 instead of an arginine (R) in human AVPR2 reduced the ligand binding activity to less than 10% of the standard (Tsukaguchi et al., 1995). Our study found the loss of allele 202-R in the Platyrrhini and Lemuroidea species (a leucine residue is found in both taxa). Another example is the alteration of threonine (T) to asparagine (N) at position 204 of AVPR2, which reduces the affinity for AVP and explains the unresponsiveness of cNDI patients to the antidiuretic action (Postina *et al.*, 2000). More recently, Meyerowitz et al. (2022) showed that a lysine (K) at position 100 of AVPR2 in Cercopithecoidea (Old World monkeys, including humans) promotes Mg^2+^ insensitivity. In the paralog positions of other oxytocinergic system receptors (OXTR, AVPR1a, and AVPR1b), the aspartic acid (D) is conserved, including in Cercopithecoidea (Meyerowitz *et al.*, 2022). The authors assert that the sensitivity or insensitivity of the oxytocin receptors to magnesium (Mg^2+^) has critical evolutionary implications (Meyerowitz *et al.*, 2022). 

The stimulation of the AVPR2-AVP complex controls the membrane presence of the protein AQP2, while ubiquitination regulates its exposure in the membrane through specific domains in the AQP2 C-terminal region (Kamsteeg et al., 2006). Positions 230 to 243 in the AQP2 C-terminal domain are critical for signaling and lysosomal degradation and promote internalization due to this leucine-rich region (Frick et al., 2014). Our study found that the co-evolving site at position 230 in AQP2 contains a tryptophan (W) residue in Platyrrhini and the ancestral residue leucine (L) in Catarrhini. The ubiquitination target site 270 is located near the co-evolving site at position 269 serine (S) in Platyrrhini and threonine (T) in Catarrhini. Böselt et al. (2009) studied the relationship between the climate in the habitats of marsupials, rodents, and aquatic mammal species. They discovered that arid-adapted marsupials had high urine osmolality levels similar to those of other desert mammals. Böselt *et al.* (2009) stated that the increased basal function of AVPR2 observed in several marsupials may be responsible for their ability to concentrate urine and thus maintain water and electrolyte balance under conditions of limited water supply. However, the authors did not identify a specific amino acid that would cause constitutive activity when substituted. They suggested that some unknown combination of amino acids in marsupial AVPR2 increases the receptor’s basal function (Böselt *et al.*, 2009).

Our study investigated primate species (*e.g*. *Sapajus libidinosus*) that live in the dry Brazilian biomes, the Caatinga and Cerrado. However, our correlation analysis between primates’ AVPR2 taxon-specific variants, with potential functional and evolutionary significance, and the climate in the niches/biomes that the investigated primate species inhabit did not find statistical significance, unlike Böselt et al. (2009). We did not find any differences in these amino acids at sites between *Homo sapiens* and other apes. This result suggests that previously known differences in water turnover and water/energy ratio between *Homo sapiens* and other apes (Pontzer et al., 2021) cannot be attributed to the genes analyzed here, at least in terms of the sites we emphasized.

Some potential limitations to our study need to be considered. Firstly, the 19 bioclimatic variables we utilized are based on the species’ current climate and geographical distribution. However, they do not encompass long-term climatic conditions that could substantially influence evolutionary dynamics. Secondly, it is essential to note that the taxon-specific variants we identified are just a subset of a more extensive and yet-to-be-fully-explored adaptive epistatic repertoire for each taxon. This constatation implies that a single amino acid change’s impact relies on other factors within the same pathway or in other pathways. Additionally, a mutation can affect the receptor’s affinity for a ligand, disrupt stability and functionality, and necessitate additional compensatory mutations within a specific adaptive context with selective pressures (Domingo et al., 2019). Lastly, the molecules under investigation in our study also involve other pathways, such as glucocorticoid hormone metabolism, highlighting their pleiotropic nature. Thus, selective pressures may not solely arise from climatic factors alone. For example, Taheri et al. (2022) showed that male and female mice submitted to the insulin tolerance test, simulating hypoglycemia as a stress factor, AVPR2 transcripts increased in the hypothalamus and decreased in the pituitary but just in males. These findings highlight the association between the AVP-AVPR2 complex and the activation of the HPA system, which stimulates cortisol release. This physiological response, with pronounced sex differences, plays a crucial role in regulating metabolic stress. 

Finally, primates have high cognitive abilities that can lead to innovations with adaptive gain in climatically hostile environments. For example, chimpanzees living in the savanna biome of Senegal have adapted their behavior to regulate their body temperature during heat stress, such as soaking in streams during the transitional period between the dry and wet seasons (Pruetz, 2021). Individuals of the *Sapajus libidinosus* species living in Brazilian dry biomes use tools to access food and water that would otherwise be inaccessible. They also frequently demonstrate innovative behaviors and social learning, which ensures the transgenerational transmission of novelty as a form of culture (Falótico and Ottoni, 2013). For example, a female *Sapajus libidinosus* was observed using a twig to reach accumulated rainwater in a tree hole, while others used their hands and mouth to manipulate orchids’ pseudobulbs and the liquid endosperm of palm nuts (Castro et al., 2017). Besides, despite the remarkable adaptations promoted by culture in *Homo sapiens*, the lack of significant differences with the other apes in this study highlights the complex nature of the subject. No cultural aspect was considered in our analysis.

In conclusion, understanding the intricate genetic basis of phenotypic adaptations, such as the ability to adapt to varying water supplies, is challenging. This challenge is further compounded by the capacity of the primates to find cultural solutions to environmental challenges and by the presence of epistatic and pleiotropic effects in the genetic system studied here. Nevertheless, our study aimed to contribute to this understanding by exploring, for the first time, the evolutionary role of *AVPR2* taxon-specific variants in primates. Despite evidence for the functional significance of some of these variants, their evolutionary context remains uncertain, so our results should be cautiously approached.

## Supplementary material

The following online material is available for this article:

Data 1 - Material and methods.

Table S1 - 
*AVPR2* Primates species analyzed.

Table S2 - Primer sets designed to flank coding regions of *AVPR2*.

Table S3 - Bioclimatic variables

Table S4 - 
*AVP* Primates species analyzed.

Table S5 - 
*AQP2* Primates species analyzed.

Table S6 - Evolutionary maximum likelihood parameters for *AVPR2.*

Table S7 - MEME evolution test.

Table S8 - Predicted SLiMS for AVPR2.

Table S9 - Sites in covariation between AVP^a^, AVPR2, and AQP2

Figure S1 - Schematic representation of the AVPR2 receptor (a) with its three coding regions.

Figure S2 - Graphic representation of the predicted SLiMs.

Figure S3 - (A) The plot of the results of the phylogenetic Partial Least Square (pPLS) analysis that assesses the association between Block 1 (variables considering twelve AVPR2 sites) and Block 2 (19 Bioclimatic variables). (B) The plot of the results of the phylogenetic Partial Least Square (pPLS) analysis that assesses the association between Block 1 (variables considering twelve AVPR2 sites) and Block 2 (Principal Components PC1 and PC2).

Figure S4 - (A) The plot of the results of the phylogenetic Partial Least Square (pPLS) analysis that assesses the association between Block 1 (coevolving sites, AVPR2/AQP2/AVP complete coding sequence) and Block 2 (19 Bioclimatic variables); (B) The plot of the results of the phylogenetic Partial Least Square (pPLS) analysis that assesses the association between Block 1 (coevolving sites, AVPR2/AQP2/AVP complete coding sequence) and Block 2 (PC1 and PC2).
