# Supplementary material for: Exploring the diversity of AVPR2 in Primates and its evolutionary implications
Source: Genet Mol Biol. 2023 Nov 3;46(3):e20230045. doi: 10.1590/1678-4685-GMB-2023-0045 (PMC10626583; doi:10.1590/1678-4685-GMB-2023-0045)
Supplement: Data 1 - [file 1415-4757-GMB-46-3-e20230045-s1.pdf]

Genetics and Molecular Biology

DOI: https://doi.org/10.1590/1678-4685-GMB-2023-0045

Supplementary Material to "Exploring the diversity of AVPR2 in Primates and its evolutionary implications"

Data 1 - Materials and Methods

Primate sampling and sequencing of AVPR2

The current project is registered in two official Brazilian systems: the Biodiversity Authorization and Information System (SISBIO; protocol number 57039; 09/01/2017), which permits the collection of biological material from conservation units for research, and SISGEN (National System for the Management of Genetic Heritage and Associated Traditional Knowledge; protocol number AF00ED5; 27/09/2018), an instrument to assist the Genetic Heritage Management Council (CGEN) in the management of genetic heritage and associated traditional knowledge. The "Serra da Capivara" National Park (SCNP) field activities, including the collection of biological samples, were also authorized by SISBIO (protocol numbers: 48323-1, 05/05/2015 and 59019-1, 23/06/2017). The State Environmental Institute (INEA), linked to the State Department of the Environment of "Rio de Janeiro," also authorized the genetic studies using Rio de Janeiro's Primatology Center (CPRJ) specimens (administrative procedure E-07 / 002.12978 / 2017). The Animal Ethics Committee of the "Universidade Federal do Rio Grande do Sul" approved the project "The molecular evolution of behavioral genes in primates" on November 25, 2014. The Animal Ethics Committee of the "Universidade de São Paulo" approved the project (CEUA/IP/USP #3036140715). Our studies with these samples comply with the principles proposed by the American Society of Primatologists for the ethical treatment of non-human primates (https://www.asp.org/society/resolutions/Ethical TreatmentOfNonHumanPrimates.cfm).

Blood or fecal 38 Platyrrhini species (Table S1) were collected in CPRJ and SCNP, respectively. CPRJ, where animals are kept in captivity without public access, stretches over 239.54 ha with a forest cover of 95%. The CPRJ is located in Guapimirim, RJ, Brazil (22o27'S-22o32'S, 42o50'W-42o56'W). The SCNP was created in 1979 with the primary goal of protecting a rich pre-Colombian archaeological heritage. Due to its historical and cultural value, it was declared by UNESCO in 1991 a World Heritage Site. The SCNP has 135,000 hectares and is located in the

northeastern Brazilian state of Piauí (8º50'S-42º33'W) (ICMBio, 2020). SCNP has some populations of the Platyrrhini species *Sapajus libidinosus*, and since these animals live free, the collection of biological samples was non-invasive (feces).

DNA extraction was performed using Qiagen DNeasy Blood and Tissue Kit® protocol according to the manufacturer's instructions. Primers were designed to cover all exonic regions with Primer3 software (Untergasser *et al.*, 2012) (Table S2). PCR products were obtained using initial denaturation at 94°C for 5 minutes, followed by 40 cycles of the 30s at 94°C, 30s at each primer annealing temperature (Table S2), and an extension step of 45s at 72°C, followed by final extension for 10 minutes at 72°C. Amplification success was verified by electrophoresis on a 2% agarose gel dyed with GelRed™. The amplicon was examined under UV light with a low mass 100bp molecular marker as a control. PCR products were purified using exonuclease I and alkaline phosphatase (Amersham Biosciences) according to the indicated protocol. Amplified fragments were sequenced using an external service provider with Sanger sequencing using ABI PRISM® 3730XL DNA Analyzer (Thermo Fisher Scientific, 96 capillary types) and checked through the Codon Code Aligner software (version 4.0). Sequences were deposited in GenBank under accession code OP289481-OP289520. Our analyses also included forty AVPR2 coding sequences retrieved from the public database GenBank (https://www.ncbi.nlm.nih.gov/) (Table S1) and twenty-five AVP coding sequences (Table S3). The AVP gene is located on chromosome 20 in humans. It contains three main functional domains, producing the neurohormone with nine amino acids, Neurophysin 2 peptide, and a C-terminal glycopeptide called Copeptin. The AVP gene also encodes a signal peptide. We also obtained the 28 AQP2 coding sequences (Table S4). Sequences were aligned using the MUSCLE (Edgar, 2004) algorithm implemented in Aliview software (Larsson, 2014).

Evolutionary analysis of the Primates AVPR2.

The AVPR2 The AVPR2 coding sequences data set includes 45 Platyrrhini, 25 Catarrhini, and 6 Strepsirrhini species, plus two outgroup species (order Scandentia and Dermoptera), which are considered Primates basal taxa. To estimate evolutionary rates in Primates AVPR2, we performed Phylogenetic Analysis by MaximumLikelihood (PAML). A consensus phylogenetic tree based on neutral molecular markers is obtained from the Timetree database (https://timetree.org/; Kumar *et al.*, 2017). This phylogeny agrees with the proposed by Perelman *et al.* (2011) and later revised by Schneider and Sampaio (2015). We also used evolutionary tests to estimate the rate of

non-synonymous to synonymous substitutions defined by the equation ω = dN/dS (dN = non-synonymous and dS = synonymous).

The ω values serve as an indicator of negative selection (ω < 1), neutral or relaxed selection (ω ≈ 1), or positive selection (ω > 1) acting on sites and/or specific phylogenetic tree branches (Nei and Kumar, 2000; Yang, 2006). To avoid false-positive results, only genomic regions with the total covered in all species analyzed were considered in evolutionary analyses.sequences data set includes 45 Platyrrhini, 25 Catarrhini, and 6 Strepsirrhini species, plus two outgroup species (order Scandentia and Dermoptera), which are considered Primates basal taxa.

To estimate evolutionary rates in Primates AVPR2, we performed Phylogenetic Analysis by Maximum Likelihood (PAML). A consensus phylogenetic tree based on neutral molecular markers is obtained from the Timetree database (https://timetree.org/; Kumar *et al.*, 2017). This phylogeny agrees with the proposed by Perelman *et al.* (2011) and later revised by Schneider and Sampaio (2015). We also used evolutionary tests to estimate the rate of non-synonymous to synonymous substitutions defined by the equation ω = dN/dS (dN = non-synonymous and dS = synonymous). The ω values serve as an indicator of negative selection (ω < 1), neutral or relaxed selection (ω ≈ 1), or positive selection (ω > 1) acting on sites and/or specific phylogenetic tree branches (Nei and Kumar, 2000; Yang, 2006). To avoid false-positive results, only genomic regions with the total covered in all species analyzed were considered in evolutionary analyses.

Two approaches are used to estimate evolutionary rates using the *codeml* program in PAML v4.9 (Yang, 2007): across sites and along branches in the phylogeny. To determine ω variation across sites in the alignment, we fit neutral (M1a and M8a) or positive selection (M2a and M8) models to the data using maximum likelihood. To assess the statistical significance of the tested model and determine if positive selection models are significantly more likely to fit our data than neutral models, we performed Likelihood Ratio Tests (LRTs) accordingly model pairs: M1a vs. M2a with df = 2, and M8a vs. M8 with df = 1 (Yang, 1998, 2007). M2a and M8 models admit positive selection. In cases in which tests indicate models of positive selection were significantly more likely, we used post hoc Bayes Empirical Bayes (BEB) to infer individual sites with a high probability (P > 0.95) of being under positive selection (Yang *et al.*, 2005).

We also infer evolutionary rates using the Mixed Effects Model of Evolution (MEME; Murrell *et al.*, 2012) to detect pervasive and episodic positive selection. The MEME test uses also ω as metric. This approach is implemented in Hyphy (https://www.hyphy.org/; Pond *et al.*, 2005). MEME allows the distribution of ω to vary from site to site and branch to branch at a site (Murrell *et al.*, 2012). MEME is also a conceptual advance over the first generation of

random effects models designed to detect episodic selection (called “branch-site models” in the literature), since MEME treats the selective class on each branch as a random effect that is marginalized in the likelihood calculation (Murrell *et al.*, 2012). Spielman *et al.* (2019) showed that site-level methods such as MEME tend to be conservative on biological data, so they recommend a p-value ≤ 0.1 for statistical significance thresholds

The Platyrrhini branch was marked as a foreground branch allowing ω to vary within, contrasting with other branches in the tree, considered a background branch with fixed ω. To infer ω variation along Primate branches, we used Branch-sites models implemented in PAML codeml, which allow ω to vary among branches and sites, considering Bayes Empirical Bayes (BEB) approach to (Clade model C) (Yang, 2007). We performed an LRT to compare the neutral model (M2a_rel) and the selection model (Clade C) by their log-likelihood with df = 1 (Weadick and Chang, 2012). To assess the effect of residue changes, we calculate the Grantham Score (Grantham, 1974) prediction of the amino acids changes between Homo sapiens and *Callithrix jacchus*, considering the chemical properties, including polarity and molecular volume, characterized into classes of increasing chemical dissimilarity: conservative (0-50), moderately conservative (51-100), moderately radical (101-150), or radical (≥151).

Short linear motif predicting.

Short linear motifs (SLiM) are short stretches of adjacent amino acids in protein sequences and are vital for regulating cell physiology by mediating protein-protein interactions. We predicted the existence of SLiMs using the Eukaryotic Linear Motif (ELM) web server (http://elm.eu.org). ELM is the most comprehensive repository of experimentally validated SLiMs (Dinkel *et al.*, 2014; Van Roey *et al.*, 2014).

Since these predictions can introduce false positives (Teyra *et al.*, 2017), we only considered SLiMs in disordered regions (IDRs) of the proteins, supported by experimental evidence in ELM. We just considered SLiMs with the probability of them being found at random ≤ 6%.

Bioclimatic data vs. Primates taxon-specific AVPR2 variants.

The seventy-six Primates species with available genetic data were analyzed according to their geographic distribution. Environmental variables of the regions where the species are geographically distributed were also obtained from the IUCN database (https://www.iucnredlist.org/). Bioclimatic variables were obtained through the Worldclim database (http://www.worldclim.com/version2) with a 30’ resolution (1 Km^2). The two species that were not considered were *Homo sapiens* because of its cosmopolitan distribution and *Saguinus ochraceus* (also considered

a subspecies; *Saguinus martinsi ochraceus*; Rylands and Mittermeier, 2009), which does not have consistent distribution data for the analysis. From the spatial distribution, the average of each one of the 19 bioclimatic variables was estimated for each of the analyzed species (Table S5) using *rgeos* (v.0.5-9; Bivand and Rundel, 2020) and *rgdal* (v.1.5-28, Bivand *et al.*, 2020) packages in the R environment.

The multivariate phylogenetic comparative methods to analyze the level of correlation between the climatic/ecological data and relevant AVPR2 sites were performed. The Phylogenetic Partial Least Square (pPLS) analysis, a test based on covariance matrix, with accounting phylogenetic relationship among taxa (Adams and Felice, 2014), was implemented in the R environment with geomorph package (v.4.0.1; Baken *et al.*, 2021). This methodology explored covariation among genetic data and 19 bioclimatic variables (Table S5). Furthermore, Principal Component Analysis (PCA) was performed to convert these 19 possibly correlated bioclimatic variables into the number smallest of artificial variables (PCs) corresponding to most of the observed variance.

Co-evolution analyses.

As seen in the Introduction section, AVPR2, activated by AVP, enhances AQP2 levels, promoting water and electrolyte homeostasis. Thus, we evaluated the co-evolution process of these molecules in Primates species available in public databases through Fastcov (Shen and Li, 2016). Fastcov provides a reliable and effective approach to identifying covariant pairs, as well as detecting multiple covariance and sequence classification, which are most helpful in studying the point and compensatory mutations caused by natural selection (Shen and Li, 2016). The analyses were performed considering the three molecules only in species with data available for the three genes (Tables S1, S3 and S4). We considered covariant sites with a degree ≥ 0.95.

The correlation of the coevolving amino acids considering AVP, AVPR2, and AQP2 with the bioclimatic data were tested using the same procedures described in the previous item.

REFERENCES

Adams DC and Felice RN (2014) Assessing trait covariation and morphological integration on phylogenies using evolutionary covariance matrices. PLoS One 9:e94335.

Baken EK, Collyer ML, Kaliontzopoulou A and Adams DC (2021) geomorph v4.0 and gmShiny: Enhanced analytics and a new graphical interface for a comprehensive morphometric experience. Methods Ecol Evol 12:2355–2363.

Bivand R and Rundel C (2020) rgeos: Interface to geometry engine—open source ('GEOS'). R package version 0.5-3. https://CRAN.R-project.org/package=rgeos (accessed 01 December 2021).

Bivand R, Keitt T and Rowlingson B (2020) rgdal: Bindings for the 'geospatial' data abstraction library. R package version 1.5-16. https://CRAN.R-project.org/package=rgdal (accessed 01 December 2021).

Dinkel H, Van Roey K, Michael S, Davey NE, Weatheritt RJ, Born D, Speck T, Krüger D, Grebnev G, Kubań M *et al.* (2014) The eukaryotic linear motif resource ELM: 10 years and counting. Nucleic Acids Res 42:D259–D266.

Edgar RC (2004) MUSCLE: Multiple sequence alignment with high accuracy and high throughput. Nucleic Acids Res 32:1792–1797.

Grantham R (1974) Amino acid difference formula to help explain protein evolution. Science 185:862–864.

Kumar S, Stecher G, Suleski M and Hedges SB (2017) TimeTree: A resource for timelines, timetrees, and divergence times. Mol Biol Evol 34:1812–1819.

Larsson A (2014) AliView: A fast and lightweight alignment viewer and editor for large datasets. Bioinformatics 30:3276–3278.

Murrell B, Wertheim JO, Moola S, Weighill T, Scheffler K and Pond SLK (2012) Detecting individual sites subject to episodic diversifying selection. PLoS Genet 8:e1002764.

Nei M and Kumar S (2000) Molecular evolution and phylogenetics. Oxford University Press, Oxford, 665 p.

Omasits U, Ahrens CH, Müller S and Wollscheid B (2014) Protter: Interactive protein feature visualization and integration with experimental proteomic data. Bioinformatics 30:884–886.

Pond SLK, Frost SDW and Muse SV (2005) HyPhy: Hypothesis testing using phylogenies. Bioinformatics 21:676–679.

Perelman P, Johnson WE, Roos C, Seuánez HN, Horvath JE, Moreira MAM, Kessing B, Pontius J, Roelke M, Rumpler Y *et al*. (2011) A molecular phylogeny of living primates. PLoS Genet 7:e1001342.

Rylands A and Mittermeier R (2009) The diversity of the New World Primates (Platyrrhini): An annotated taxonomy. In: Garber PA, Estrada A, Bicca-Marques JC, Heymann EW, Strier KB (eds) South American Primates, Springer, New York, pp 23–54.

Schneider H and Sampaio I (2015) The systematics and evolution of New World primates – A review. Mol Phylogenet Evol 82:348–357.

Shen W and Li Y (2016) A novel algorithm for detecting multiple covariance and clustering of biological sequences. Sci Rep 6:30425.

Spielman SJ, Weaver S, Shank SD, Magalis BR, Li M and Kosakovsky Pond SL (2019) Evolution of Viral Genomes: Interplay Between Selection, Recombination, and Other Forces. In: Anisimova M (ed) Evolutionary Genomics: Statistical and Computational Methods. Springer, New York, pp 427–468.

Teyra J, Huang H, Jain S, Guan X, Dong A, Liu Y, Tempel W, Min J, Tong Y, Kim PM *et al*. (2017) Comprehensive analysis of the human SH3 domain family reveals a wide variety of non-canonical specificities. Structure 25:1598-1610.e3.

Untergasser A, Cutcutache I, Koressaar T, Ye J, Faircloth BC, Remm M and Rozen SG (2012) Primer3—new capabilities and interfaces. Nucleic Acids Res 40:e115.

Van Roey K, Uyar B, Weatheritt RJ, Dinkel H, Seiler M, Budd A, Gibson TJ and Davey NE (2014) Short linear motifs: Ubiquitous and functionally diverse protein interaction modules directing cell regulation. Chem Rev 114:6733–6778.

Weadick CJ and Chang BSW (2012) An improved likelihood ratio test for detecting site-specific functional divergence among clades of protein-coding genes. Mol Biol Evol 29:1297–1300.

Yang Z (1998) Likelihood ratio tests for detecting positive selection and application to primate lysozyme evolution. Mol Biol Evol 15:568–573.

Yang Z (2006) Computational Molecular Evolution. 1st edition. Oxford Academic, Oxford.

Yang Z (2007) PAML 4: Phylogenetic analysis by maximum likelihood. Mol Biol Evol 24:1586–1591.

Yang Z, Wong WSW and Nielsen R (2005) Bayes empirical bayes inference of amino acid sites under positive selection. Mol Biol Evol 22:1107–1118.

Spatial data reference:

Alonso AC, Jerusalinsky L, Mittermeier RA and Régis T (2018) Mico saterei. The IUCN Red List of Threatened Species 2018:e.T42692A17933401.

Ancrenaz M, Gumal M, Marshall AJ, Meijaard E, Wich SA and Husson S (2016) Pongo pygmaeus (errata version published in 2018). The IUCN Red List of Threatened Species 2016:e.T17975A123809220.

Andriaholinirina N, Baden A, Blanco M, Chikhi L, Cooke A, Davies N, Dolch R, Donati G, Ganzhorn J, Golden C *et al*. (2014a) Lemur catta. The IUCN Red List of Threatened Species 2014:e.T11496A62260437.

Andriaholinirina N, Baden A, Blanco M, Chikhi L, Cooke A, Davies N, Dolch R, Donati G, Ganzhorn J, Golden C *et al*. (2014b) Microcebus murinus. The IUCN Red List of Threatened Species 2014:e.T13323A16113348.

Andriaholinirina N, Baden A, Blanco M, Chikhi L, Cooke A, Davies N, Dolch R, Donati G, Ganzhorn J, Golden C *et al*. (2014c) Propithecus coquereli. The IUCN Red List of Threatened Species 2014:e.T18355A16115770.

Andriaholinirina N, Baden A, Blanco M, Chikhi L, Cooke A, Davies N, Dolch R, Donati G, Ganzhorn J, Golden C *et al*. (2014d) Varecia variegata. The IUCN Red List of Threatened Species 2014:e.T22918A16121857.

Bezerra B, Bicca-Marques J, Miranda J, Mittermeier RA, Oliveira L, Pereira D, Ruiz-Miranda C, Valença Montenegro M, da Cruz M and do Valle RR (2018) Callithrix jacchus. The IUCN Red List of Threatened Species 2018:e.T41518A17936001.

Bleisch W and Richardson M (2008) Rhinopithecus bieti. The IUCN Red List of Threatened Species 2008:e.T19597A8986243.

Bleisch B, Geissmann T, Manh Ha N, Rawson B and Timmins RJ (2008) Nomascus leucogenys. The IUCN Red List of Threatened Species 2008:e.T39895A10272040.

Boeadi and Steinmetz R (2008) Galeopterus variegatus. The IUCN Red List of Threatened Species 2008:e.T41502A10479343.

Boubli J. 2018. Cacajao melanocephalus. The IUCN Red List of Threatened Species 2018:e.T70558706A17975783.

Boubli J, de Azevedo R and Rohe F (2018) Plecturocebus caligatus. The IUCN Red List of Threatened Species 2018:e.T41552A17973006.

Boubli J-P, Di Fiore A, Rylands AB and Mittermeier RA (2008) Alouatta discolor. The IUCN Red List of Threatened Species 2008:e.T43912A10836686.

Boubli J, Alves SL, Buss G, Carvalho A, Ceballos-Mago N, Alfaro JL, Messias M, Mittermeier RA, Palacios E, Ravetta A *et al.* (2018) Sapajus apella. The IUCN Red List of Threatened Species 2018:e.T39949A70611337.

Brockelman W and Geissmann T (2008) Hylobates lar. The IUCN Red List of Threatened Species 2008:e.T10548A3199623.

Butynski TM, Bearder S and De Jong Y (2008) Otolemur garnettii. The IUCN Red List of Threatened Species 2008:e.T15644A4945217.

Cornejo F (2008) Callimico goeldii. The IUCN Red List of Threatened Species 2008:e.T3564A9947398.

Cuarón AD, Morales A, Shedden A, Rodríguez-Luna E and de Grammont PC (2008) Cebus capucinus ssp. capucinus. The IUCN Red List of Threatened Species 2008:e.T43934A10841636.

de la Torre S and Rylands AB (2008) Cebuella pygmaea. The IUCN Red List of Threatened Species 2008:e.T41535A10493764.

de Oliveira MM and Kierulff MCM (2008) Alouatta ululata. The IUCN Red List of Threatened Species 2008:e.T918A13094890.

Defler TR and García J (2012) Plecturocebus caquetensis. The IUCN Red List of Threatened Species 2012:e.T14699281A14699284.

Fruth B, Hickey JR, André C, Furuichi T, Hart J, Hart T, Kuehl H, Maisels F, Nackoney J, Reinartz G *et al*. (2016) Pan paniscus. The IUCN Red List of Threatened Species 2016:e.T15932A102331567.

Gippoliti S and Ehardt T (2008) Papio hamadryas. The IUCN Red List of Threatened Species 2008:e.T16019A5354647.

Gippoliti S and Hunter C (2008) Theropithecus gelada. The IUCN Red List of Threatened Species 2008:e.T21744A9316114.

Global Mammal Assessment Team (2008) Homo sapiens. The IUCN Red List of Threatened Species 2008:e.T136584A4313662.

Han KH, Duckworth JW and Molur S (2016) Tupaia belangeri. The IUCN Red List of Threatened Species 2016:e.T41492A22280884.

Humle T, Maisels F, Oates JF, Plumptre A and Williamson EA (2016) Pan troglodytes. The IUCN Red List of Threatened Species 2016:e.T15933A129038584.

Kierulff MCM, Mendes SL and Rylands AB (2015a) Sapajus nigritus. The IUCN Red List of Threatened Species 2015:e.T136717A70614145.

Kierulff MCM, Mendes SL and Rylands AB (2015b) Sapajus robustus. The IUCN Red List of Threatened Species 2015:e.T42697A70614762.

Kierulff MCM, Mendes SL and Rylands AB (2015c) Sapajus xanthosternos. The IUCN Red List of Threatened Species 2015:e.T4074A70615251.

Kierulff MCM, Rylands AB and de Oliveira MM (2008) Leontopithecus rosalia. The IUCN Red List of Threatened Species 2008:e.T11506A3287321.

Kierulff MCM, Rylands AB, Mendes SL and de Oliveira MM (2008) Leontopithecus chrysopygus. The IUCN Red List of Threatened Species 2008:e.T11505A3290864.

Kierulff MCM, Rylands AB, Mendes SL and de Oliveira MM (2008) Leontopithecus chrysopygus. The IUCN Red List of Threatened Species 2008:e.T11505A3290864.

Kingdon J and Butynski TM (2008) Chlorocebus aethiops. The IUCN Red List of Threatened Species 2008:e.T4233A10695029.

Kingdon J and Gippoliti S (2008) Chlorocebus sabaeus. The IUCN Red List of Threatened Species 2008:e.T136265A4267012.

Kingdon J, Butynski TM and De Jong Y (2008a) Papio anubis. The IUCN Red List of Threatened Species 2008:e.T40647A10348950.

Kingdon J, Struhsaker T, Oates JF, Hart J and Groves CP (2008b) Colobus guereza. The IUCN Red List of Threatened Species 2008:e.T5143A11116447.

Kingdon J, Struhsaker T, Oates JF, Hart J, Butynski TM, De Jong Y and Groves CP (2008c) Colobus angolensis. The IUCN Red List of Threatened Species 2008:e.T5142A11116129.

Maisels F, Bergl RA and Williamson EA (2018) Gorilla gorilla (amended version of 2016 assessment). The IUCN Red List of Threatened Species 2018:e.T9404A136250858.

Maldonado A, Guzman-Caro D, Shanee S, Defler TR and Roncancio N (2017) Aotus nancymaae (amended version of 2017 assessment). The IUCN Red List of Threatened Species 2017:e.T41540A121725532.

Marsh LK, Mittermeier RA and Rylands AB (2019) Pithecia mittermeieri . The IUCN Red List of Threatened Species 2019:e.T70610693A70610705.

Mendes SL, de Oliveira MM, Mittermeier RA and Rylands AB (2008a) Brachyteles arachnoides. The IUCN Red List of Threatened Species 2008:e.T2993A9529160.

Mendes SL, Rylands AB, Kierulff MCM and de Oliveira MM (2008b) Alouatta guariba ssp. clamitans. The IUCN Red List of Threatened Species 2008:e.T39918A10285929.

Mittermeier RA and Rohe F (2018) Mico chrysoleucos. The IUCN Red List of Threatened Species 2018:e.T39910A70616096.

Mittermeier RA, Rylands AB and Boubli J-P (2008a) Ateles paniscus. The IUCN Red List of Threatened Species 2008:e.T2283A9392691.

Mittermeier RA, Rylands AB and Boubli J (2018) Saguinus midas. The IUCN Red List of Threatened Species 2018:e.T41525A17932579.

Mittermeier RA, Boubli J-P, Subirá R and Rylands AB (2008b) Saguinus bicolor. The IUCN Red List of Threatened Species 2008:e.T40644A10348136.

Nijman V and Meijaard E (2008) Trachypithecus cristatus. The IUCN Red List of Threatened Species 2008:e.T22035A9348474.

Oates JF and Butynski TM (2008a) Mandrillus leucophaeus ssp. leucophaeus. The IUCN Red List of Threatened Species 2008:e.T12756A3378112.

Oates JF and Butynski TM (2008b) Mandrillus sphinx. The IUCN Red List of Threatened Species 2008:e.T12754A3377579.

Oates JF, Gippoliti S and Groves CP (2016) Cercocebus atys. The IUCN Red List of Threatened Species 2016:e.T136933A92247942.

Ong P and Richardson M (2008) Macaca fascicularis. The IUCN Red List of Threatened Species 2008:e.T12551A3355536.

Richardson M, Mittermeier RA, Rylands AB and Konstant B (2008) Macaca nemestrina. The IUCN Red List of Threatened Species 2008:e.T12555A3356892.

Rohe F and Boubli J (2018) Plecturocebus dubius. The IUCN Red List of Threatened Species 2018:e.T3549A17975232.

Rylands AB and Mittermeier RA (2008) Saguinus niger. The IUCN Red List of Threatened Species 2008:e.T42694A10733990.

Rylands AB and Silva Jr. JS (2008) Mico humeralifer. The IUCN Red List of Threatened Species 2008:e.T41521A10488246.

Rylands AB and Kierulff MCM (2015) Sapajus libidinosus. The IUCN Red List of Threatened Species 2015:e.T136346A70613080.

Rylands AB and Mendes SL (2018) Callithrix geoffroyi. The IUCN Red List of Threatened Species 2018:e.T3572A17936610.

Rylands AB and Mittermeier RA (2018) Callibella humilis. The IUCN Red List of Threatened Species 2018:e.T41584A70616532.

Rylands AB, Mittermeier RA and Wallace RB (2008a) Mico melanurus. The IUCN Red List of Threatened Species 2008:e.T136294A4270667.

Rylands AB, Mittermeier RA and Subirá R (2008b) Saguinus martinsi ssp. ochraceus. The IUCN Red List of Threatened Species 2008:e.T43963A10834912.

Rylands AB, Silva Jr. JS and Mittermeier RA (2008c) Mico mauesi. The IUCN Red List of Threatened Species 2008:e.T41583A10483538.

Rylands AB, Mittermeier RA and Subirá R (2014) Saguinus martinsi. The IUCN Red List of Threatened Species 2014:e.T42695A17930887.

Rylands AB, Kierulff MCM, Mendes SL and de Oliveira MM (2008) Callithrix aurita. The IUCN Red List of Threatened Species 2008:e.T3570A9949843.

Shanee S, Alves SL, Calouro AM, Lynch Alfaro J, Messias M, Ravetta A, Rohe F and Romero-Valenzuela D (2018) Aotus nigriceps. The IUCN Red List of Threatened Species 2018:e.T41542A17923573.

Shekelle M and Arboleda I (2008) Tarsius syrichta. The IUCN Red List of Threatened Species 2008:e.T21492A9289252.

Singleton I, Wich SA, Nowak M, Usher G and Utami-Atmoko SS (2017) Pongo abelii (errata version published in 2018). The IUCN Red List of Threatened Species 2017:e.T121097935A123797627.

Spielman SJ, Weaver S, Shank SD, Magalis BR, Li M and Kosakovsky Pond SL (2019) Evolution of Viral Genomes: Interplay Between Selection, Recombination, and Other Forces. In: Anisimova M (ed) Evolutionary Genomics: Statistical and Computational Methods. Springer, New York, NY, pp 427–468.

Struhsaker T (2016) Piliocolobus tephrosceles. The IUCN Red List of Threatened Species 2016:e.T18256A92660998.

Timmins RJ, Richardson M, Chhangani A and Yongcheng L (2008) Macaca mulatta. The IUCN Red List of Threatened Species 2008:e.T12554A3356486.

Veiga LM and Ferrari SF (2008) Plecturocebus moloch. The IUCN Red List of Threatened Species 2008:e.T41556A10477784.

Veiga LM, Silva Jr. JS, Ferrari SF and Rylands AB (2008a) Chiropotes utahickae. The IUCN Red List of Threatened Species 2008:e.T43892A10830166.

Veiga LM, Silva Jr. JS, Ferrari SF and Rylands AB (2008b) Chiropotes Satanas. The IUCN Red List of Threatened Species 2008:e.T39956A10297662.

Veiga LM, Ferrari SF, Kierulff CM, de Oliveira MM and Mendes SL (2008c) Callicebus personatus. The IUCN Red List of Threatened Species 2008:e.T3555A9940882.

Veiga LM, Pinto LP, Ferrari SF, Rylands AB, Mittermeier RA and Boubli J-P (2008d) Chiropotes albinasus. The IUCN Red List of Threatened Species 2008:e.T4685A11085894.

Veiga LM, Sousa MC, Jerusalinsky L, Ferrari SF, de Oliveira MM, Santos SSD, Valente MCM and Printes RC (2008e) Callicebus coimbrai. The IUCN Red List of Threatened Species 2008:e.T39954A10297332.

Wallace R, Cornejo FM and Rylands AB (2018a) Saimiri boliviensis. The IUCN Red List of Threatened Species 2018:e.T41536A17940021.

Wallace R, Martinez JL, Ferrari S and Veiga LM (2018b) Plecturocebus donacophilus. The IUCN Red List of Threatened Species 2018:e.T41548A17972581.

Wu S, Birnbaumer M and Guan Z (2008) Phosphorylation analysis of G Protein-coupled receptor by mass spectrometry: Identification of a novel phosphorylation site in V2 vasopressin receptor. Anal Chem 80:6034–6037.

Yongcheng L and Richardson M (2008) Rhinopithecus roxellana. The IUCN Red List of Threatened Species 2008:e.T19596A8985735.
