# Supplementary material for: Exploring the diversity of AVPR2 in Primates and its evolutionary implications
Source: Genet Mol Biol. 2023 Nov 3;46(3):e20230045. doi: 10.1590/1678-4685-GMB-2023-0045 (PMC10626583; doi:10.1590/1678-4685-GMB-2023-0045)
Supplement: Table S1 - [file 1415-4757-GMB-46-3-e20230045-s2.pdf]

Genetics and Molecular Biology
DOI: https://doi.org/10.1590/1678-4685-GMB-2023-0045

Supplementary Material to "Exploring the diversity of AVPR2 in Primates and its evolutionary implications"

Table S1 - *AVPR2* Primates species analyzed.

Specie	Reference	Specie	Reference
Homo sapiens	XM_006724828	*Mico melanura*	OP289498
Pan paniscus	XM_014343594.1	*Mico chrysoleucus*	OP289493
Pan troglodytes	XM_001145732.4	*Mico humeralifera*	OP289495
Gorilla gorilla	XM_004065096.2	*Callimico goeldi*	OP289491
Pongo abelii	XM_009235419.1	*Leontopithecus chrysomelas*	OP289504
Pongo pygmaeus	FJ411239.1	*Leontopithecus chrysopygas*	OP289505
Nomascus leucogenys	XM_003279300.2	*Leontopithecus rosalia*	OP289506
Hylobates lar	FJ411208.1	*Saguinus oechraceous*	OP289516
Cercocebus atys	XM_012088358.1	*Saguinus bicolor*	OP289512
Mandrillus sphinx	FJ411220.1	*Saguinus martinsi*	OP289513
Mandrillus leucophaeus	XM_012002270.1	*Saguinus niger*	OP289515
Theropithecus gelada	XM_025371777.1	*Saguinus midas*	OP289514
Papio anubis	XM_009198486.3	*Cebus capucinus*	XM_017516681.1
Papio hamadryas	FJ411237.1	*Sapajus apella*	XM_032265248.1
Macaca mulatta	XM_001088539.3	*Sapajus libidinosus*	OP289517
Macaca fascicularis	XM_005594940.2	*Sapajus xanthosternos*	OP289520
Macaca nemestrina	XM_011716796.1	*Sapajus nigritus*	OP289518
Chlorocebus aethiops	F411195.1	*Sapajus robustus*	OP289519
Chlorocebus sabaeus	XM_007993105.1	*Saimiri boliviensis*	XM_010331788.1
Piliocolobus tephrosceles	XM_023200696.1	*Cacajao melanocephalus*	OP289487
Colobus angolensis	XM_011955879.1	*Chiropotes albinasus*	OP289501
Colobus guereza	FJ411196.1	*Chiropotes satanas*	OP289502
Presbitys cristata	FJ411241.1	*Chiropotes utahickae*	OP289503
Rhinopithecus bieti	XM_017880754.1	*Pithecia mittermeier*	OP289507
Rhinopithecus roxellana	XM_010361734.1	*Callicebus moloch*	OP289511
Alouatta ululata	OP289483	*Callicebus caligatis*	OP289508
Alouatta discolor	OP289482	*Callicebus donacophilus*	OP289489
Alouatta clamitans	OP289481	*Callicebus dubius*	OP289510
Atles paniscus	OP289485	*Callicebus personatus*	OP289490
Brachyteles arachnoides	OP289486	*Callicebus coimbrai*	OP289488
Aotus nigriceps	OP289484	*Callicebus caquetensis*	OP289509
Aotus nancymaae	XM_012437907.2	*Tarsius syrichta*	XM_008071553.1
Callithrix geoffroyi	OP289494	*Lemur catta*	FJ411214.1s
Callithrix jacchus	XM_017968313.1	*Varecia variegata*	FJ411249.1
Callithrix aurita	OP289492	*Microcebus murinus*	XM_012757255.2

Specie	Reference	Specie	Reference
Cebuella pygmaea	OP289499	*Propithecus coquereli*	XM_012662037.1
Calibella humilis	OP289496	*Otolemur garnetti*	XM_003802545.2
Mico saterei	OP289500	*Galeopterus varigatus*	XM_008578396.1
Mico mauesi	OP289497	*Tupaia belangeri chinensis*	XM_006166683.2
