# Supplementary material for: Exploring the diversity of AVPR2 in Primates and its evolutionary implications
Source: Genet Mol Biol. 2023 Nov 3;46(3):e20230045. doi: 10.1590/1678-4685-GMB-2023-0045 (PMC10626583; doi:10.1590/1678-4685-GMB-2023-0045)
Supplement: Table S2 - [file 1415-4757-GMB-46-3-e20230045-s3.pdf]

Genetics and Molecular Biology
DOI: https://doi.org/10.1590/1678-4685-GMB-2023-0045

Supplementary Material to "Exploring the diversity of AVPR2 in Primates and its evolutionary implications"

Table S2 - Primer sets designed to flank whole coding regions of *AVPR2*.

**Primers 5' -> 3'**		
Exon1	V2_1F	CTTGGGCAGAGGCTGAGTC
	V2_1R	AAACCCACTGTGCCCACC
Exon2	V2_2F	TTCCCCTGCACAGCACC
	V2_2R	AAGATGAAGAGCTGGGGCAG
	V2_3F	GCARATGGTGGGCATGTA
	V2_3R	ACAGYTGCACCAGGAAGAAG
Exon3	V2_4F	TCTATGTGCTRTGCTGGGC
	V2_4R	GAAGGCAGCTGAGCTTCTCA
