# Supplementary material for: Exploring the diversity of AVPR2 in Primates and its evolutionary implications
Source: Genet Mol Biol. 2023 Nov 3;46(3):e20230045. doi: 10.1590/1678-4685-GMB-2023-0045 (PMC10626583; doi:10.1590/1678-4685-GMB-2023-0045)
Supplement: Table S3 - [file 1415-4757-GMB-46-3-e20230045-s4.pdf]

Genetics and Molecular Biology
DOI: https://doi.org/10.1590/1678-4685-GMB-2023-0045

Supplementary Material to "Exploring the diversity of AVPR2 in Primates and its evolutionary implications"

Table S3 - Values referring to the means of the respective Bioclimatic variables* according to the spatial distribution for each primate species analyzed and for the values of the two first principal components loadings.

Species	Bio1	Bio2	Bio3	Bio4	Bio5	Bio6	Bio7	Bio8	Bio9	Bio10	Bio11	Bio12	Bio13	Bio14	Bio15	Bio16	Bio17	Bio18	Bio19	PC1	PC2
Alouatta clamitans	19.92259	11.0003	59.41074	268.7596	28.94432	10.30791	18.63642	21.61185	17.68934	23.00123	16.48851	1414.5650	194.6553	62.03852	41.97059	530.2110	213.8069	474.6737	233.5018	19.337	0.78958
Alouatta discolor	25.91142	10.90285	84.39831	48.16231	32.26438	19.3144	12.94998	25.40716	26.19817	26.55172	25.37537	2098.205	334.3696	24.26297	65.46875	933.2517	100.1751	275.5592	901.5831	-30.185	-12.982
Alouatta ululata	26.52333	11.16334	82.08821	77.55158	33.55286	19.93039	13.62246	25.93251	27.03263	27.54286	25.75628	1343.43	312.0367	6.854235	102.7275	809.6749	25.83663	58.88639	546.7983	11.273	29.856
Aotus nancymaae	25.62782	9.825575	87.04416	39.45745	31.08621	19.80898	11.27722	25.66987	25.18838	26.04259	25.09397	2351.7790	274.5215	103.9342	29.77289	769.3975	354.4102	607.9567	391.8568	21.628	-15.482
Aotus nigriceps	25.68067	10.71786	85.57424	53.36701	31.74417	19.15521	12.58896	25.73302	25.18064	26.23614	24.97041	2143.2930	305.9534	43.21422	55.48913	867.0481	164.4358	487.1555	334.9468	15.778	0.14111
Ateles paniscus	26.10982	9.263629	80.58656	58.28835	32.10680	20.60621	11.50059	25.75858	26.77475	26.90712	25.47821	2227.2340	353.1483	67.92857	53.39712	948.5449	237.001	254.5932	734.8741	24.933	-0.19646

Species	Bio1	Bio2	Bio3	Bio4	Bio5	Bio6	Bio7	Bio8	Bio9	Bio10	Bio11	Bio12	Bio13	Bio14	Bio15	Bio16	Bio17	Bio18	Bio19	PC1	PC2
Brachyteles arachnoides	19.27546	10.22213	57.15389	261.4451	28.19943	10.34219	17.85724	22.29427	16.03525	22.35447	16.0007	1584.803	243.2293	49.53133	53.27849	675.0956	173.9887	650.9345	175.8814	-30.075	-1.811
Cacajao melanocephalus	26.22389	8.866677	82.96601	47.23068	31.35569	20.65613	10.69956	25.86229	26.49055	26.74035	25.58563	2956.251	359.1263	141.2158	30.51290	1018.7152	476.0462	546.5047	884.5387	36.127	-25.397
Callicebus coimbrai	24.23382	8.588555	63.54162	160.2622	30.45882	16.89909	13.55972	23.57481	24.90848	25.86629	22.06029	1088.384	173.9005	37.92607	51.06663	466.9536	132.4842	202.7609	357.9236	30.678	-0.20646
Callicebus personatus	22.04213	11.32379	64.76196	197.3015	30.69845	13.20378	17.49467	23.53367	19.39708	24.28055	19.35805	1154.898	201.8581	24.70773	66.68746	560.2613	87.21641	397.2006	87.33631	37.059	-31.397
Callimico goeldii	25.54771	10.05014	83.07592	57.04656	31.31143	19.21136	12.10007	25.69835	25.07741	26.08455	24.738550	2398.819	286.7557	104.7325	35.48324	812.2981	351.8857	639.4823	424.5831	-0.81102	0.32171
Callithrix aurita	19.79914	11.20126	60.15926	232.9546	29.04928	10.44918	18.6001	22.11131	16.7953	22.50165	16.78628	1437.295	252.7511	28.01698	68.40116	685.7837	98.1038	607.4352	99.09128	13.609	0.27367
Callithrix geoffroyi	22.82833	10.56403	65.94529	181.2663	30.71888	14.72891	15.98997	24.0584	20.47016	24.84042	20.32167	1116.561	181.9307	31.77713	59.0665	503.3941	113.9562	359.9669	116.4614	14.012	11.683
Callithrix jacchus	25.31342	11.54958	76.68814	102.8083	32.63948	17.60327	15.03621	25.09444	25.09646	26.5679	24.10658	1024.977	214.0762	5.665599	90.89048	561.1599	24.20898	149.4544	200.292	-31.121	-0.71834
Carlito syrichta	26.0072	9.155186	78.17815	77.55129	31.9203	20.11457	11.80573	26.00359	25.78288	26.95678	25.03876	2609.985	346.2717	120.0742	34.65122	930.1963	392.9121	623.5634	634.2047	28.056	-1.869
Cebuella pygmaea	25.81106	10.00342	86.14153	48.9701	31.41942	19.77061	11.64881	25.94037	25.24008	26.30201	25.13701	2314.012	296.7712	72.00553	43.77818	841.213	254.7587	593.0032	322.3059	19.979	-0.78775

Species	Bio1	Bio2	Bio3	Bio4	Bio5	Bio6	Bio7	Bio8	Bio9	Bio 10	Bio 11	Bio 12	Bio 13	Bio 14	Bio 15	Bio 16	Bio 17	Bio 18	Bio 19	PC1	PC2
Cebus imitator	23.5033	11.91039	78.55953	75.73115	30.98376	15.49018	15.49358	23.5654	22.66126	24.24475	22.49534	1546.639	225.9381	25.40941	59.8253	613.8668	109.3575	407.1445	173.3991	16.813	0.5684
Cercocebus atys	25.86108	11.15815	74.75027	114.6133	33.04316	18.10912	14.93404	25.03712	25.76396	27.34281	24.62935	2390.157	507.2053	19.14859	84.3848	1255.901	94.43617	330.9132	943.6536	0.79588	0.76143
Chiropotes albinasus	25.74041	11.34388	83.85335	53.01289	32.37481	18.73848	13.63633	25.47378	25.63126	26.36572	25.10466	2105.197	331.1602	24.06205	65.83055	934.5094	103.1641	349.3452	601.8318	30.106	-0.41124
Chiropotes satanas	26.1611	10.64309	86.37716	49.91615	32.28079	19.94991	12.33088	25.70463	26.44752	26.84229	25.64792	1914.966	365.832	27.95784	78.02617	978.4515	978.4515	171.5147	889.9227	14.969	11.713
Chiropotes utahickae	25.5623	12.02327	86.67801	44.1254	32.34754	18.45299	13.89455	25.1794	25.66379	26.13022	25.05019	1992.009	324.4162	22.90391	69.00093	915.7239	96.19778	273.1382	752.8174	-18.147	32.834
Chlorocebus aethiops		13.87147	72.15724	184.2399	33.41478	13.99126	19.42352	23.70055	23.27478	26.40163	21.93801	850.1795	179.9773	7.833963	92.68077	459.4132	33.82636	140.6672	315.1124	-0.069012	42.927
Chlorocebus sabaeus	27.0847	12.59663	67.3974	208.012	35.95065	17.03127	18.91937	26.29569	25.86949	29.84573	22.49534	495.34	1087.19	268.5145	2.211989	111.4899	674.209	14.65496	150.9264	-0.78594	0.64572
Colobus angolensis	23.5033	11.91039	78.55953	75.73115	30.98376	15.49018	15.49358	23.5654	22.66126	24.24475	22.49534	1546.639	225.9381	25.40941	59.8253	613.8668	109.3575	407.1445	173.3991	-0.0062986	0.70896
Colobus guereza	24.40215	10.67716	80.22623	91.7112	30.74187	17.29338	13.44849	23.75663	24.3407	25.64301	23.44168	1510.62	236.7711	22.65922	62.50426	641.9015	95.17523	297.0449	440.4113	2.718	-26.596
Galeopterus variegatus	25.10924	8.921215	87.47781	36.07152	30.30173	20.10491	10.19682	24.84185	25.09081	25.55491	24.70882	2653.529	320.9537	120.7285	31.29368	892.0296	406.554	657.0183	778.5191	0.12145	-0.75876

Species	Bio1	Bio2	Bio3	Bio4	Bio5	Bio6	Bio7	Bio8	Bio9	Bio 10	Bio 11	Bio 12	Bio 13	Bio 14	Bio 15	Bio 16	Bio 17	Bio 18	Bio 19	PC1	PC2
Gorilla gorilla	24.34433	8.654353	76.88455	86.70712	29.53435	18.29097	11.24338	24.2921	23.58743	23.20333	23.20333	1748.38	288.2708	25.7139	57.54616	701.4117	117.926	518.5577	247.8258	-13.073	17.288
Hylobates lar	24.74791	9.91701	56.62085	251.8209	32.45486	14.88378	17.57107	25.92439	21.53611	27.29823	21.20565	1252.684	246.4989	4.598363	84.20567	642.6577	24.98034	394.0711	33.30631	-28.202	37.225
Lemur catta	23.83907	14.30853	64.73333	282.1693	34.37056	12.31131	26.67866	26.67866	20.4332	26.72694	19.99171	701.2054	172.1631	5.764618	103.512	454.9334	21.97273	422.5092	27.28967	-0.78278	-11.399
Leontopithecus chrysomelas	23.36304	7.887769	65.15534	154.1429	29.25511	17.15737	12.09774	24.45784	21.91905	25.05169	21.27996	1245.114	146.3275	68.09673	26.79255	396.4913	234.9598	339.0653	263.8085	-31.021	-0.34167
Leontopithecus chrysopygus	20.29345	11.92506	61.16217	262.1178	29.74222	10.25151	19.49071	23.16728	17.23607	23.17576	16.87217	1249.653	195.8998	37.48223	53.1797	539.2918	139.8142	529.548	158.0923	-16.968	0.16364
Leontopithecus rosalia	22.7847	9.166042	57.64842	218.8829	30.94051	15.08056	15.85995	25.23302	20.22135	25.53607	20.13313	1164.482	172.4551	30.65776	52.73997	476.4435	108.5573	452.247	110.907	38.039	-30.447
Macaca fascicularis	26.26729	9.465969	87.45869	33.8894	31.68127	20.84035	10.84092	26.11039	26.28343	26.65321	25.86667	3032.709	336.2656	166.2695	22.69853	939.7891	550.5565	760.3318	818.1148	-51.262	0.9418
Macaca mulatta	18.9932	11.03419	40.61759	597.1659	32.12204	4.485156	27.63688	22.82865	14.19796	25.79406	11.15408	1134.559	252.8071	11.00398	95.8295	654.8205	47.00085	443.8209	63.82715	38.039	-30.447
Macaca nemestrina	26.26751	9.465558	87.45794	33.8946	31.68131	20.8408	10.84052	26.11057	26.28364	26.65344	25.86688	3032.729	336.2691	166.2652	22.69898	939.8009	550.5508	760.3389	818.1466	20.598	-0.67279
Mandrillus leucophaeus	25.13943	8.685754	75.47262	94.22838	30.61042	19.1	11.51042	23.96642	25.64043	26.25544	23.87054	2501.412	406.7167	16.56244	69.56837	1152.151	94.22584	367.6202	1136.491	0.31467	-0.7283

Species	Bio1	Bio2	Bio3	Bio4	Bio5	Bio6	Bio7	Bio8	Bio9	Bio 10	Bio 11	Bio 12	Bio 13	Bio 14	Bio 15	Bio 16	Bio 17	Bio 18	Bio 19	PC1	PC2
Mandrillus sphinx	24.33591	7.842082	71.62082	104.5152	29.18226	18.23477	10.94749	24.43418	23.26018	25.38587	22.85707	1876.576	339.7723	16.21251	66.87829	791.643	82.40234	580.8731	170.4647	29.354	-0.22626
Mico chrysoleucus	26.92475	9.507057	88.27566	43.49407	32.40505	21.64269	10.76236	26.47895	27.09237	27.53719	26.46928	2221.15	294.5411	66.81908	46.5888	850.3558	228.3103	411.4982	848.8097	11.772	0.48083
Mico humeralifera	26.66011	9.553422	84.65968	56.95421	32.45586	21.1726	11.28326	26.12933	27.05068	27.47337	26.09698	2194.289	319.5535	59.2023	52.45452	899.8586	218.1456	319.7247	881.5663	-22.858	0.64897
Mico humilis	26.68086	10.29947	91.23462	34.15026	32.28268	20.99379	11.28889	26.3606	26.63053	27.13794	26.33392	2516.955	351.2459	44.31349	53.93442	1025.249	176.8524	513.1415	845.135	1.189	-16.111
Mico mauesi	26.7994	9.748763	87.2351	46.26188	32.37884	21.20868	11.17016	26.35898	26.84286	27.44924	26.31824	2257.246	317.4606	54.69656	52.81141	915.0608	199.7814	399.5686	902.7016	-17.733	0.54372
Mico melanurus	25.09108	12.46066	71.10155	163.6268	33.17438	15.62607	17.54831	26.27433	23.03831	26.58723	22.74901	1449.996	241.0893	19.35475	65.95979	669.2842	79.95181	476.0901	107.8025	-0.47537	27.703
Mico saterei	26.95702	9.543975	87.75774	44.94367	32.46894	21.59521	10.87373	26.52238	27.01202	27.59359	26.47831	2249.668	298.9484	63.1055	48.08673	865.5117	227.3129	411.9971	858.7262	21.958	0.8537
Microcebus murinus	25.11331	13.65067	67.5764	221.048	34.73915	14.48347	20.25568	27.14626	22.20631	27.27536	22.03089	984.5371	274.3825	3.017231	117.6759	681.9447	13.19295	536.7581	14.71373	27.449	0.0086331
Nomascus leucogenys	22.14261	9.124309	51.58371	325.614	30.08603	12.38265	17.70338	25.09085	18.02536	25.34938	17.64222	1820.151	408.9499	5.206815	96.94104	1075.763	33.33861	919.1588	37.75718	28.652	-0.048579
Otolemur garnettii	23.47216	10.41381	69.30627	158.6744	30.61507	15.57283	15.04224	24.37696	21.81135	25.13472	21.30326	955.026	203.6532	6.847151	90.01992	515.7638	29.00233	309.1541	45.7787	-0.44942	17.433

Species	Bio1	Bio2	Bio3	Bio4	Bio5	Bio6	Bio7	Bio8	Bio9	Bio 10	Bio 11	Bio 12	Bio 13	Bio 14	Bio 15	Bio 16	Bio 17	Bio 18	Bio 19	PC1	PC2
Pan paniscus	24.85542	9.566993	85.71255	50.6269	30.47571	19.31352	11.16219	24.63094	24.65548	25.5005	24.25163	1862.543	232.6529	71.89686	32.90778	634.6827	271.3826	472.8195	377.4652	29.487	-0.17834
Pan troglodytes	23.62204	10.41318	84.5753	62.1339	29.60168	17.19923	12.40245	23.22128	23.62774	24.40859	22.9277	1600.654	214.3494	36.89761	48.0799	590.7082	144.6079	350.2152	417.4777	-12.419	35.269
Papio anubis	25.5331	12.62813	73.05138	169.0768	33.82382	16.11868	17.70514	24.91555	24.53239	27.76609	23.62006	965.3689	196.7159	8.704966	96.32806	502.9532	36.84977	163.9197	257.1618	-16.173	-0.62105
Papio hamadryas	24.52494	12.32378	61.81056	266.0361	34.39082	14.2566	20.13482	25.61581	23.20487	27.61617	21.23686	392.5308	81.20105	4.833977	72.45169	190.5985	25.30028	119.8475	54.29184	-17.177	15.905
Piliocolobus tephrosceles	21.31433	10.351	80.43508	62.95011	27.49078	14.53939	12.95139	21.29882	20.77164	21.98527	20.59419	1092.985	191.5231	8.873767	71.31923	465.2722	44.47332	280.3518	90.12309	1.168	-0.98722
Pithecia mittermeier	25.57163	11.76457	82.92169	66.06889	32.44724	18.09148	14.35576	25.6213	25.01085	26.25655	24.69437	2010.669	315.3052	23.85762	65.40091	890.2858	104.0657	403.8473	350.0891	-0.094787	-0.24243
Plectorucebus donacophilus	25.60046	11.03915	73.68614	134.9561	32.47685	17.47202	15.00482	26.5109	23.80451	26.8083	23.66483	1732.234	280.6629	31.92583	64.29505	782.5408	119.5122	585.8724	148.673	30.701	-0.52316
Plecturocebus caligatus	26.87915	9.233933	89.51858	36.52875	32.20247	21.88862	10.31385	26.54481	26.99642	27.37744	26.5086	2311.57	296.6172	73.53744	44.11499	853.632	253.5492	433.2323	812.0515	-0.87293	33.656
Plecturocebus caquetensis	25.40493	8.92001	77.55068	65.54452	30.91195	19.40939	11.50257	25.00169	26.12601	26.12608	24.51115	3533.646	420.6602	136.3298	32.74063	1235.74	489.015	497.0945	1062.454	-24.123	3.587
Plecturocebus dubius	25.66959	10.82041	84.74362	61.63311	31.72192	18.91965	12.80227	25.87551	24.84816	26.26593	24.79601	2011.698	286.9006	28.16944	58.04272	823.226	118.8218	517.1355	173.9857	-22.535	0.070319

Species	Bio1	Bio2	Bio3	Bio4	Bio5	Bio6	Bio7	Bio8	Bio9	Bio10	Bio11	Bio12	Bio13	Bio14	Bio15	Bio16	Bio17	Bio18	Bio19	PC1	PC2
Plecturocebus moloch	25.54042	11.9741	84.24491	48.55109	32.52515	18.21826	14.30689	25.18095	25.55	26.1399	24.97782	2019.382	329.094	18.62122	69.6333	926.6477	81.3622	319.281	716.6182	11.312	10.342
Pongo abelii	23.2332	9.030451	85.12417	45.40962	28.84066	18.22378	10.61688	22.89339	23.19745	23.8578	22.78149	2508.598	23.8578	414.3625	29.85859	852.3623	414.3625	645.2681	676.6607	15.863	-48.344
Pongo pygmaeus	26.82827	9.167543	90.52528	27.6264	31.93116	21.80903	10.12213	26.76654	26.73559	27.13769	26.52808	2905.951	321.7506	145.7854	25.41004	903.4451	484.3992	776.4601	783.3904	3.949	-25.791
Presbitys cristata	25.10924	8.921210	87.47779	36.07150	30.30173	20.10491	10.196814	24.84185	25.09081	25.55491	24.70882	2653530	320.9537	120.7286	31.29364	892.0295	406.5543	657.0187	778.5188	2.639	-16.386
Propithecus coquereli	26.23406	12.90579	75.30035	148.3285	34.51198	17.38826	17.12372	27.18533	24.25524	27.62345	24.13826	1527.466	441.8646	2.943002	124.0464	1072.407	12.15873	656.7926	12.84189	0.6278	28.778
Rhinopithecus bieti	7.8381	10.217	33.11138	745.1793	22.61966	-81.320830	30.751740	15.99873	-1.712514	16.71508	-1.71349	809.4084	163.1739	5.487645	84.81716	432.3413	21.9877	419.0246	21.9877	-12.88	-37.887
Rhinopithecus roxellana	4.481405	10.69874	39.01672	605.8241	18.14172	-9.246196	27.38791	11.47831	-2.169586	11.49906	-3.059004	737.2586	140.6728	8.427351	72.29802	361.9246	36.85653	361.6834	44.84107	-12.688	-48.216
Saguinus bicolor	26.67583	8.805087	84.66724	49.99439	32.14831	21.74834	10.39997	26.27076	26.89016	27.39195	26.20164	2321.555	309.0292	80.03531	44.30625	880.0728	272.5719	388.7146	853.5151	30.078	-0.70694
Saguinus martinsi	26.81326	9.174367	81.25184	63.68696	32.77318	21.48423	11.28895	26.35264	27.34559	27.73564	26.22961	2444.757	388.2819	76.93458	55.46891	1068.753	266.2021	310.1712	932.8326	3.319	-0.34858
Saguinus midas	26.07272	9.25303	80.42419	58.21509	32.07587	20.56655	11.50932	25.73231	26.75446	26.86582	25.43349	2226.801	353.2284	67.6183	53.14533	946.1758	236.1181	250.4489	722.0587	24.637	-0.1991

Species	Bio1	Bio2	Bio3	Bio4	Bio5	Bio6	Bio7	Bio8	Bio9	Bio 10	Bio 11	Bio 12	Bio 13	Bio 14	Bio 15	Bio 16	Bio 17	Bio 18	Bio 19	PC1	PC2
Saguinus niger	25.89096	11.0627	86.2966	51.34895	32.24074	19.40593	12.83482	25.40607	26.14424	26.58689	25.34534	1939.181	346.4955	27.77794	73.51424	945.7244	105.5366	197.0647	853.6294	18.684	11.281
Saimiri boliviensis	24.15908	10.92215	81.51753	84.89145	30.53894	17.02751	13.51143	24.6522	23.03353	24.93983	22.95686	2033.63	291.5785	49.93533	54.7263	824.4312	179.3372	588.7275	212.8269	0.47062	-0.49986
Sapajus apella	25.84732	10.4559	81.94425	64.45348	32.14705	19.34086	12.80619	25.68536	25.79655	26.5970	25.05091	2064.6370	333.3682	41.18179	62.22345	918.5579	153.7016	326.4442	623.1233	17.924	0.47963
Sapajus libidinosus	24.47031	12.66429	74.53114	116.4778	32.53222	15.46202	17.0702	24.8257	23.30162	25.63984	22.91609	1384.243	257.0426	7.245375	83.51437	700.0792	30.9582	365.2549	127.9469	-0.75825	20.896
Sapajus nigritus	19.57286	11.66074	59.32096	274.3919	29.05522	9.384223	19.671	21.61247	16.91698	22.67311	16.05016	1485.869	215.5693	58.26997	46.71736	584.3301	203.1119	539.3975	220.8207	-32.021	-12.532
Sapajus robustus	22.80985	10.593	66.0525	180.7509	30.69697	14.68801	16.00895	24.03	20.44419	24.80737	20.30008	1112.912	182.0027	31.16393	59.50694	503.13	111.9672	361.1359	114.5448	-21.347	11.361
Sapajus xanthosternos	22.90564	10.78889	69.58771	150.3068	30.10976	14.67324	15.43652	23.70992	21.51534	24.35321	20.78913	827.6221	141.6427	18.84793	67.4232	380.4563	67.54935	263.7167	107.6302	-17.954	0.55514
Theropithecus gelada	18.08649	15.76768	79.12173	138.3605	27.85305	7.880056	19.97299	17.53297	16.90572	19.90774	16.60206	908.2423	246.2694	8.919448	106.9597	576.0123	36.67606	186.7987	199.3847	-46.655	12.559
Tupaia belangeri	20.73655	8.859625	48.38264	365.6801	29.63664	10.43709	19.19954	23.81884	16.42476	24.6462	15.77835	1620.236	335.7828	9.76355	86.54201	897.5517	44.0472	646.473	62.65626	-27.726	-0.61909
Varecia variegata	19.77063	11.33523	62.5052	250.1515	28.29953	10.17111	18.12842	22.40535	17.44704	22.43564	16.47032	1869.949	328.5901	45.81047	72.4966	928.2717	169.1773	903.2251	203.7708	-23.313	-18.413

*(BIO1-Annual Mean Temperature, BIO2-Mean Diurnal Range(Mean of monthly (max temp-min temp), BIO3-Isothermality(BIO2/BIO7)(×100), BIO4-Temperature Seasonality(standard deviation×100), BIO5-Max Temperature of Warmest Month, BIO6-Min Temperature of Coldest Month, BIO7-Temperature Annual Range (BIO5-BIO6), BIO8-Mean Temperature of Wettest Quarter, BIO9-Mean Temperature of Driest Quarter, BIO10-Mean Temperature of Warmest Quarter, BIO11-Mean Temperature of Coldest Quarter, BIO12-Annual Precipitation, BIO13-Precipitation of Wettest Month, BIO14-Precipitation of Driest Month, BIO15-Precipitation Seasonality (Coefficient of Variation), BIO16-Precipitation of Wettest Quarter, BIO17-Precipitation of Driest Quarter, BIO18-Precipitation of Warmest Quarter, BIO19-Precipitation of Coldest Quarter)
