# Supplementary material for: Exploring the diversity of AVPR2 in Primates and its evolutionary implications
Source: Genet Mol Biol. 2023 Nov 3;46(3):e20230045. doi: 10.1590/1678-4685-GMB-2023-0045 (PMC10626583; doi:10.1590/1678-4685-GMB-2023-0045)
Supplement: Table S4 - [file 1415-4757-GMB-46-3-e20230045-s5.pdf]

Genetics and Molecular Biology
DOI: https://doi.org/10.1590/1678-4685-GMB-2023-0045

Supplementary Material to "Exploring the diversity of AVPR2 in Primates and its evolutionary implications"

Table S4 - *AVP* Primates species analyzed.

Specie	Reference	Specie	Reference
Aotus nancymaae	XM_012454959.1	*Otolemur garnettii*	XM_012804054.1
Callithrix jacchus	XM_002747402.2	*Pan paniscus*	XM_034947667.1
Carlito syrichta	XM_021707521.1	*Pan troglodytes*	XM_001160259.5
Cebus imitator	XM_017504777.2	*Papio anubis*	XM_021921193.2
Cercocebus atys	XM_012052854.1	*Piliocolobus tephrosceles*	XM_023220620.1
Chlorocebus sabaeus	XM_008019230.2	*Pongo abelii*	XM_002830097.4
Homo sapiens	NM_000490	*Rhinopithecus bieti*	XM_017872530.1
Hylobates moloch	XM_032142548.1	*Rhinopithecus roxellana*	XM_010378980.2
Macaca fascicularis	XM_005568487.2	*Saimiri boliviensis*	XM_039479651.1
Macaca mulatta	XM_001115061.4	*Sapajus apella*	XM_032294908.1
Macaca nemestrina	XM_011741311.2	*Theropithecus gelada*	XM_025400042.1
Microcebus murinus	XM_012755316.1	*Trachypithecus francoisi*	XM_033185854.1
Nomascus leucogenys	XM_003277950.3	*Tupaia belangeri chinensis*	XM_006163955.1
