# Supplementary material for: Exploring the diversity of AVPR2 in Primates and its evolutionary implications
Source: Genet Mol Biol. 2023 Nov 3;46(3):e20230045. doi: 10.1590/1678-4685-GMB-2023-0045 (PMC10626583; doi:10.1590/1678-4685-GMB-2023-0045)
Supplement: Table S5 - [file 1415-4757-GMB-46-3-e20230045-s6.pdf]

Genetics and Molecular Biology
DOI: https://doi.org/10.1590/1678-4685-GMB-2023-0045

Supplementary Material to "Exploring the diversity of AVPR2 in Primates and its evolutionary implications"

Table S5 - *AQP2* Primates species analyzed.

**Specie**	**Reference**	**Specie**	**Reference**
Aotus nancymaae	XM_012441421.1	*Nomascus leucogenys*	XM_003252170.2
Callithrix jacchus	XM_002752444.5	*Otolemur garnettii*	XM_003793622.2
Cebus imitator	XM_017545804.2	*Pan paniscus*	XM_003825854.4
Cercocebus atys	XM_012048341.1	*Pan troglodytes*	XM_016923442.2
Chlorocebus sabaeus	XM_008003189.2	*Papio anubis*	XM_009180698.4
Colobus angolensis	XM_011936154.1	*Piliocolobus tephrosceles*	XM_023211179.2
Galeopterus variegatus	XM_008582946.1	*Pongo abelii*	XM_002823203.4
Gorilla gorilla	XM_004053097.1	*Propithecus coquereli*	XM_012651371.1
Homo sapiens	NM_000486.	*Rhinopithecus bieti*	XM_017894818.1
Hylobates moloch	XM_032143889.1	*Rhinopithecus roxellana*	XM_010385371.2
Macaca fascicularis	XM_015430894.1	*Saimiri boliviensis*	XM_003939145.3
Macaca mulatta	XM_015151628.2	*Sapajus apella*	XM_032294039.1
Macaca nemestrina	XM_011760158.2	*Theropithecus gelada*	XM_025402907.1
Mandrillus leucophaeus	XM_011964704.1	*Trachypithecus francoisi*	XM_033222140.1
Microcebus murinus	XM_012761866.1	*Tupaia belangeri chinensis*	XM_006168189.2
