# Supplementary material for: Exploring the diversity of AVPR2 in Primates and its evolutionary implications
Source: Genet Mol Biol. 2023 Nov 3;46(3):e20230045. doi: 10.1590/1678-4685-GMB-2023-0045 (PMC10626583; doi:10.1590/1678-4685-GMB-2023-0045)
Supplement: Table S6 - [file 1415-4757-GMB-46-3-e20230045-s7.pdf]

Genetics and Molecular Biology
DOI: https://doi.org/10.1590/1678-4685-GMB-2023-0045

Supplementary Material to "Exploring the diversity of AVPR2 in Primates and its evolutionary implications"

Table S6 - Evolutionary maximum likelihood parameters (NsSites) estimated for the *AVPR2* gene in Primates plus outgroup Scandentia and Dermoptera.

Site Model	dN/dS	Estimated parameters	ℓ	*p* value
M1a: neutral	0.1929	p0= 0.86011, (p1= 0.13989)	-7423.964486	
		ω0= 0.06160, ω1= 1.00000		
M2a: selection	0.1929	p0=0.86011, p1= 0.07034, (p2= 0.06955)	-7423.964487	M1 *vs* M2a
		ω0= 0.06160, ω1= 1.00000, ω2=1.00000		p=1
M7: neutral	0.1583	p = 0.24495 q = 1.27553	-7403.755487	M7 *vs* M8
				p<0.001
M8a: beta	0.1501	p0= 0.95051 , (p1= 0.04949) , p= 0. 34881	-7398.384578	M8a *vs* M8
		q = 2.82784, ω= 1.00000		***p*=0.0008**
M8:beta &ω	0.1660	p0= 0.98635, (p1= 0.01365), p= 0.29952	-7392.808866	
		q= 1.90795, **ω= 2.62643**		

*p_0 = proportion of sites where ω < 1; p_1 = proportion of sites where ω = 1; p_2 = proportion of sites where ω > 1 (selection models only); $\omega_0 < 1$ (negative selection), $\omega_1 = 1$ (neutral selection), $\omega_2 > 1$ (positive selection). Likelihood ratio tests were performed between neutral models (M1a-Nearly Neutral, and M7 - Beta) and models that identify positive selection and/or relaxation of functional constraints (M2a – Selection, and M8 – Beta + Selection); Comparisons of M1a *vs* M2a and M7 *vs* M8 have two degrees of freedom (df = 2). Parentheses indicate fixed parameters.
