# Supplementary material for: Exploring the diversity of AVPR2 in Primates and its evolutionary implications
Source: Genet Mol Biol. 2023 Nov 3;46(3):e20230045. doi: 10.1590/1678-4685-GMB-2023-0045 (PMC10626583; doi:10.1590/1678-4685-GMB-2023-0045)
Supplement: Table S7 - [file 1415-4757-GMB-46-3-e20230045-s8.pdf]

Genetics and Molecular Biology
DOI: https://doi.org/10.1590/1678-4685-GMB-2023-0045

Supplementary Material to "Exploring the diversity of AVPR2 in Primates and its evolutionary implications"

Table S7 - MEME evolution test.

Sites	LRT	*p*-value	Branches under selection	MEME LogL
190	5.20	0.03	5.00	-68.79
250	4.10	0.06	2.00	-36.72
346	3.44	0.08	3.00	-38.05
