# Supplementary material for: Exploring the diversity of AVPR2 in Primates and its evolutionary implications
Source: Genet Mol Biol. 2023 Nov 3;46(3):e20230045. doi: 10.1590/1678-4685-GMB-2023-0045 (PMC10626583; doi:10.1590/1678-4685-GMB-2023-0045)
Supplement: Table S8 - [file 1415-4757-GMB-46-3-e20230045-s9.pdf]

Genetics and Molecular Biology
DOI: https://doi.org/10.1590/1678-4685-GMB-2023-0045

Supplementary Material to "Exploring the diversity of AVPR2 in Primates and its evolutionary implications"

Table S8 - Prediced SLiMS for AVPR2.

ELM category	Aminoacid position	Number ofspecieswhere the motif was predicted	Amino acid pattern	Probability score*
LIG_SH3_3	8-14	29	(+xX[P]xXP)	0.00
	11-17	31	(+xX[P]xXP)	0.00
	14-20	50	(+xX[P]xXP)	0.04
	234-240	2	(+xx[V]xxP)	0.06

*Provided by the ELM, representing the probability of it being found randomly.
