# Supplementary material for: Exploring the diversity of AVPR2 in Primates and its evolutionary implications
Source: Genet Mol Biol. 2023 Nov 3;46(3):e20230045. doi: 10.1590/1678-4685-GMB-2023-0045 (PMC10626583; doi:10.1590/1678-4685-GMB-2023-0045)
Supplement: Table S9 - [file 1415-4757-GMB-46-3-e20230045-s10.pdf]

Genetics and Molecular Biology
DOI: https://doi.org/10.1590/1678-4685-GMB-2023-0045

Supplementary Material to "Exploring the diversity of AVPR2 in Primates and its evolutionary implications"

Table S9 - Sites in covariation between AVP[a], AVPR2, and AQP2.

	Sites in co-evolution[b]	
**AVP[a]**	**AVPR2**	**AQP**
5-M	74-I 100-K 202-R 204-T 244-P 249-R 302-L 305-A	269-T
89-Q 112-T	74-I 100-K 202-R 204-T 244-P 249-R 252-R 302-L 305-A 345-G	230-L 269-T
111-M 112-T	74-I 100-K 202-R 204-T 244-P 249-R 252-R 302-L 305-A 345-G	230-L 269-T
5-T	74-M 100-D 202-L 204-A 302-R 305-P	269-S
89-H 112-I	74-M 100-D 202-L 204-A 244-T 249-P 252-H 302-R 302-P 345-R	230-W 269-S
111-V 112-I	74-M 100-D 202-L 204-A 244-T 249-P 302-R 302-P 345-R	230-W 269-S

[a] 1-19 amino acid sequence is the signal peptide, 20-28 is the neurohormone AVP, 32-124 is the Neurophisin-2, and 126-164 is the Copeptin peptide.All molecules are encoded by the *AVP* gene.

[b] The amino acid combinations vary according to the Primates clades (see Figure S2).
