# Supplementary material for: Exploring the diversity of AVPR2 in Primates and its evolutionary implications
Source: Genet Mol Biol. 2023 Nov 3;46(3):e20230045. doi: 10.1590/1678-4685-GMB-2023-0045 (PMC10626583; doi:10.1590/1678-4685-GMB-2023-0045)
Supplement: Figure S3 - [file 1415-4757-GMB-46-3-e20230045-s13.pdf]

Genetics and Molecular Biology
DOI: https://doi.org/10.1590/1678-4685-GMB-2023-0045

Supplementary Material to "Exploring the diversity of AVPR2 in Primates and its evolutionary implications"

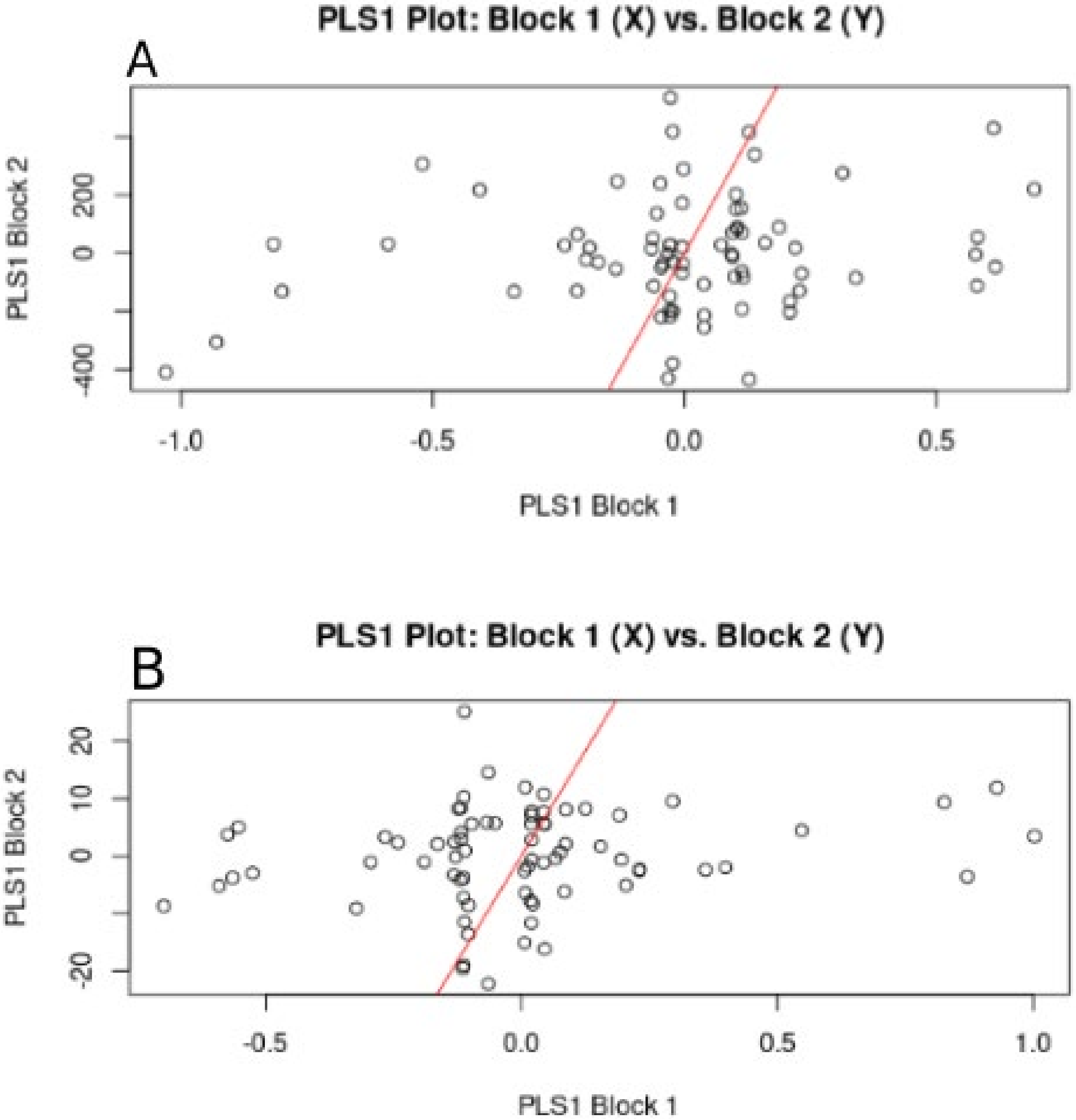

Figure S3 - (A) The plot of the results of the phylogenetic Partial Least Square (pPLS) analysis that assesses the association between Block 1 (variables considering twelve AVPR2 sites) and Block 2 (19 Bioclimatic variables). (B) The plot of the results of the phylogenetic Partial Least Square (pPLS) analysis that assesses the association between Block 1 (variables considering twelve AVPR2 sites) and Block 2 (Principal Components PC1 and PC2).
